# Histone lysine demethylases in breast cancer: molecular mechanisms, biological functions, and therapeutic intervention

**DOI:** 10.1186/s12943-025-02512-6

**Published:** 2025-12-23

**Authors:** Anqi Wang, Dianjun Qi, Yi Ma, Mozhi Wang, Haoran Dong, Chenxin Wang, Yingfan Zhang, Zheyuan Zhang, Lingwei Li, Jiayi Xu, Litong Yao, Yingying Xu

**Affiliations:** 1 Department of Breast Surgery, the First Hospital of China Medical University, Shenyang, 110001 China; 2 Department of General Practice, the First Hospital of China Medical University, Liaoning Province, Shenyang, 110001 China

**Keywords:** Lysine demethylases, Epigenetic regulation, Biological functions, Therapeutic intervention, Breast cancer

## Abstract

Breast cancer is a highly heterogeneous disease characterized by diverse molecular subtypes and complex pathogenesis. Recent advances in epigenetics have unveiled the crucial roles of lysine demethylases (KDMs) in modulating gene expression and chromatin dynamics, thereby influencing breast cancer progression, including metastasis, and therapeutic resistance. KDMs, which remove methyl groups from histone lysine residues, are mainly categorized into seven subfamilies (KDM1-7) based on their catalytic mechanisms and substrate specificities. Meanwhile, each subfamily exhibits distinct roles in breast cancer, ranging from transcriptional regulation and chromatin remodeling to interactions with non-histone proteins. Notably, KDMs exhibit subtype-specific functions in breast cancer. KDMs are also implicated in various hallmarks of breast cancer, including DNA damage response, cell cycle regulation, stemness maintenance, metabolic reprogramming, and modulation of the tumor microenvironment. KDMs represent promising targets for overcoming therapeutic resistance in breast cancer. Inhibitors targeting KDMs have shown potential to enhance the efficacy of endocrine therapy, chemotherapy, and targeted therapy by modulating oncogenic signaling pathways. The KDM family members are intricately involved in the molecular pathogenesis of breast cancer, offering a rich landscape for therapeutic intervention. This review summarizes the multifaceted molecular mechanisms and biological functions of KDMs in breast cancer, highlighting their potential as therapeutic targets.

## Introduction

Breast cancer is the most common malignancy among women worldwide, posing a significant threat to public health. Breast cancer is highly heterogeneous in terms of complex pathogenesis, characterized by genetic mutations, chromatin alterations of oncogenic signaling pathway, and cross-talking with tumor microenvironment [1, 2]. Based on the expression status of estrogen receptor (ER), progesterone receptor (PR), and human epidermal growth factor receptor 2 (HER2), breast cancer is classified into three subtypes: hormone receptors positive (HR +), Her2 positive (HER2 +), and Triple negative breast cancer (TNBC). For therapeutically oriented classification, tumors are stratified by hormone-receptor expression and Ki67-measured proliferation into luminal A-like, luminal B-like, HER2-overexpressing (non-luminal) and basal-like subtypes. Among these, the basal-like subtype exhibits a high Ki67 index and phenotypic concordance with TNBC [3, 4].

Epigenetic modification has emerged as critical regulators to drive breast cancer progression. The epigenetic modification, including DNA methylation, histone modification, and non-coding RNA regulation, can dynamically regulate gene transcription without changing the underlying DNA sequence. Histone lysine specific methylases (KMTs) and demethylases (KDMs) identified as the epigenetic enzymes have garnered considerable attention due to their pivotal roles in modulating gene transcription, DNA replication, and DNA damage repair via alteration of chromatin dynamics [5].

**Fig. 1:**
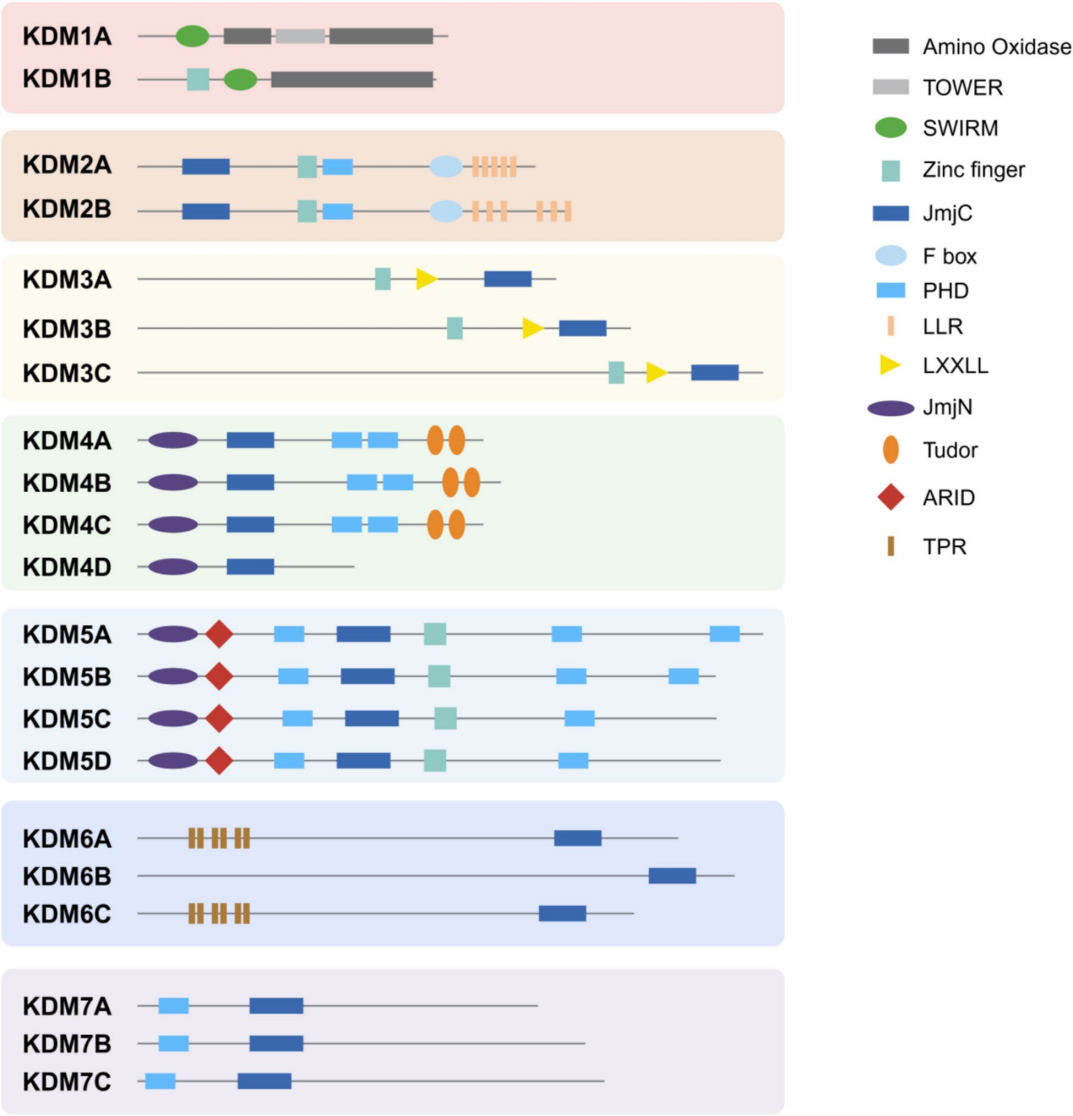
KDM family proteins carrying structure domains. The distinct KDM family members contain their conserved structure domains. KDM1 members contain Zinc finger, SWIRM, and Amino oxidase; KDM2 members contain JmjC, Zinc finger, PHD, F box, and LLR; KDM3 enzymes contain zinc finger, LXXLL, and Jmjc; KDM4 enzymes contain JmjN, JmjC, PHD, and Tudor; KDM5 enzymes contain JmjN, ARID, PHD, JmjC, and Zinc finger; KDM6 enzymes contain TPR and JmjC; KDM7 enzymes contain PHD and JmjC. The representative protein domain structures on KDM enzymes are indicated

**Fig. 2:**
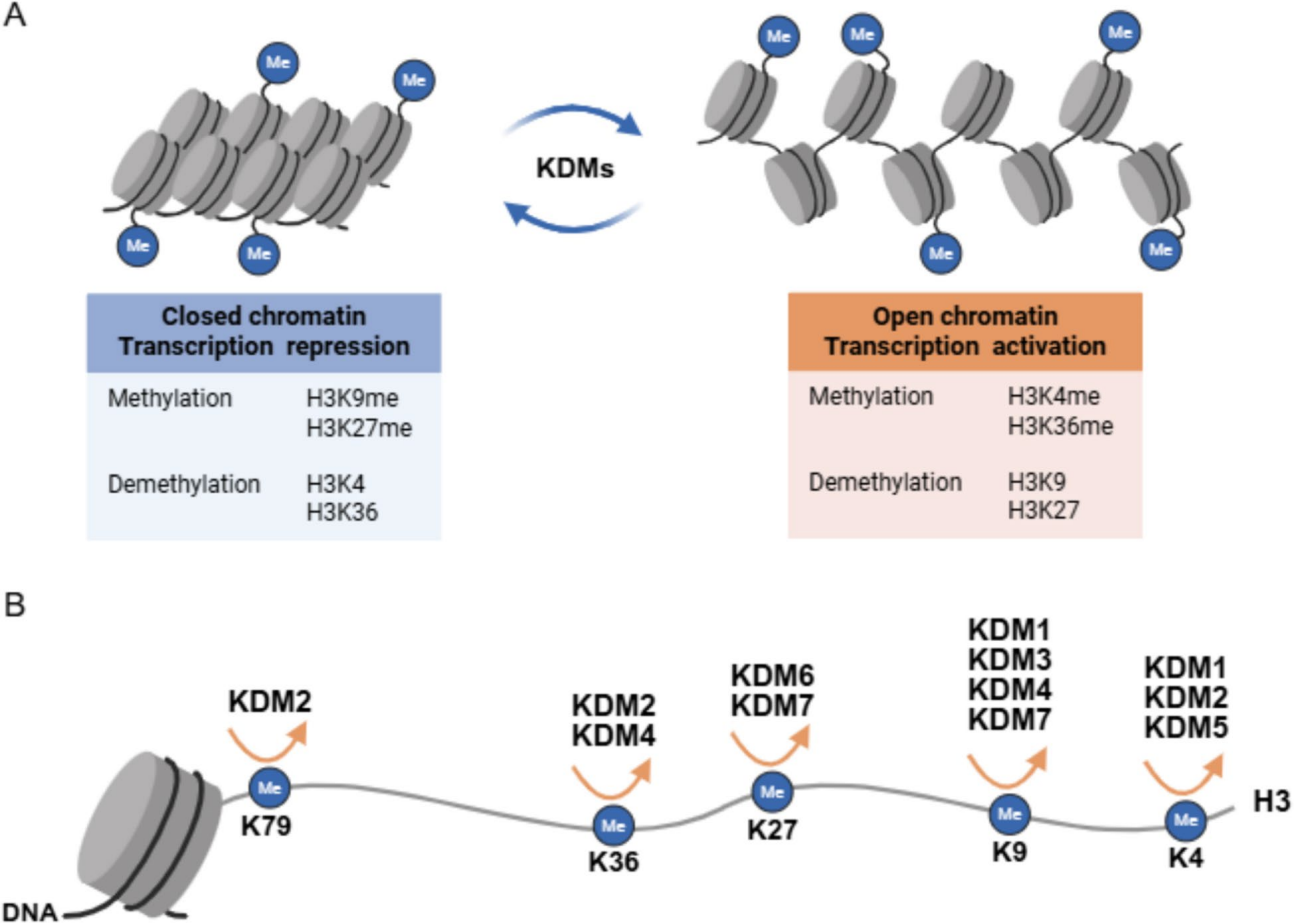
Schematic of KDM enzymes associated main histone lysine substrates on chromatin. **A** KDM enzymes are involved in regulation of gene transcription to maintain closed chromatin and open chromatin via removing corresponding histone lysine methylation. **B** KDM family proteins recognize and bind the histone tails and then catalyze hydroxylation of methyl groups on the relevant histone lysine substrates

**Fig. 3:**
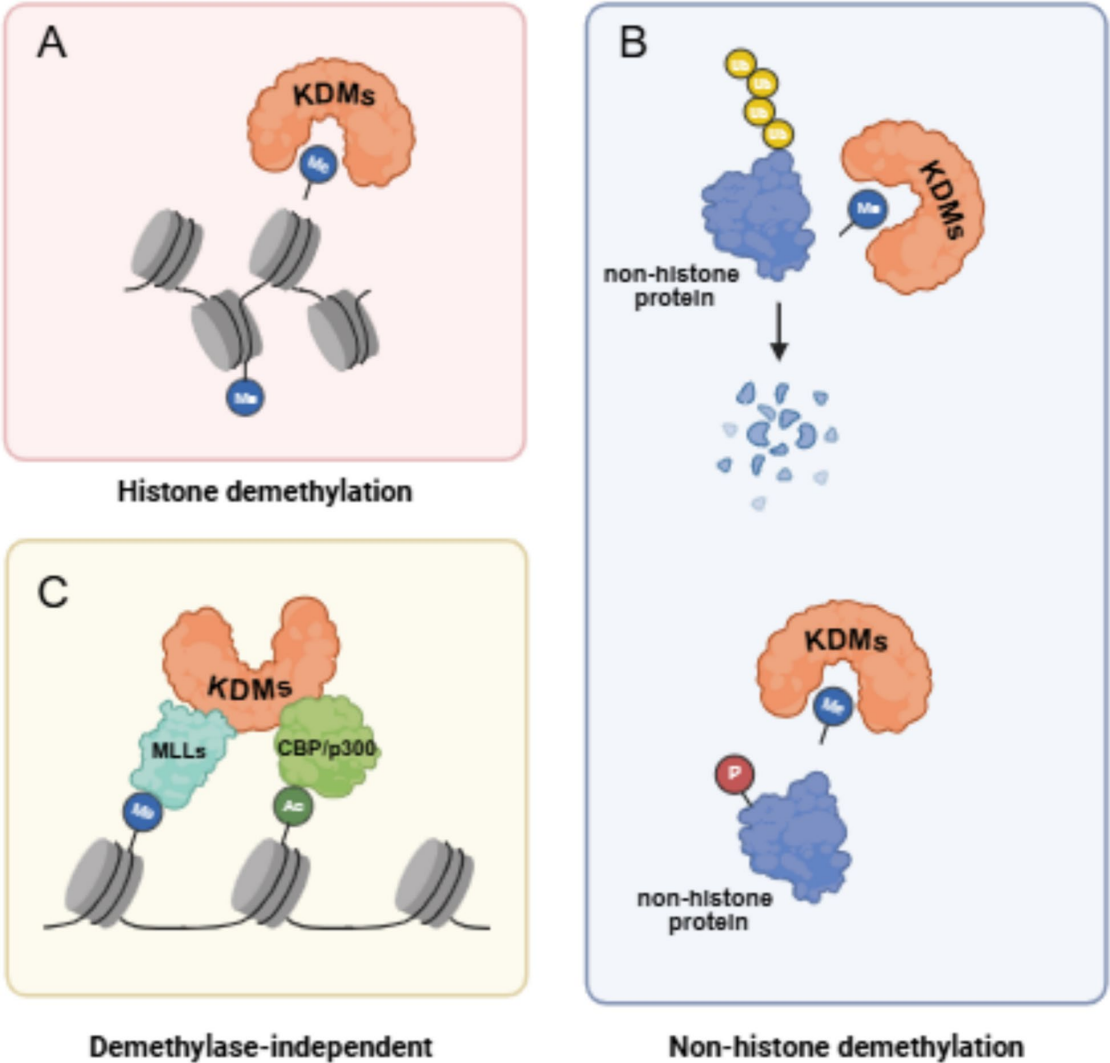
The Schematic illustration of distinct mechanisms underlying the functions of KDM enzymes in breast cancer. (**A**) KDM enzymes recognize histone tails in chromatin and catalyze hydroxylation of methyl groups. (**B**) KDM enzymes recognize and associate with non-histone proteins as substrates and then demethylate these proteins. (**C**) KDM family proteins are involved in distinct histone modifications with the assistance of co-regulator complex such as MLLs or CBP/p300 independent on their demethylase activity

**Fig. 4:**
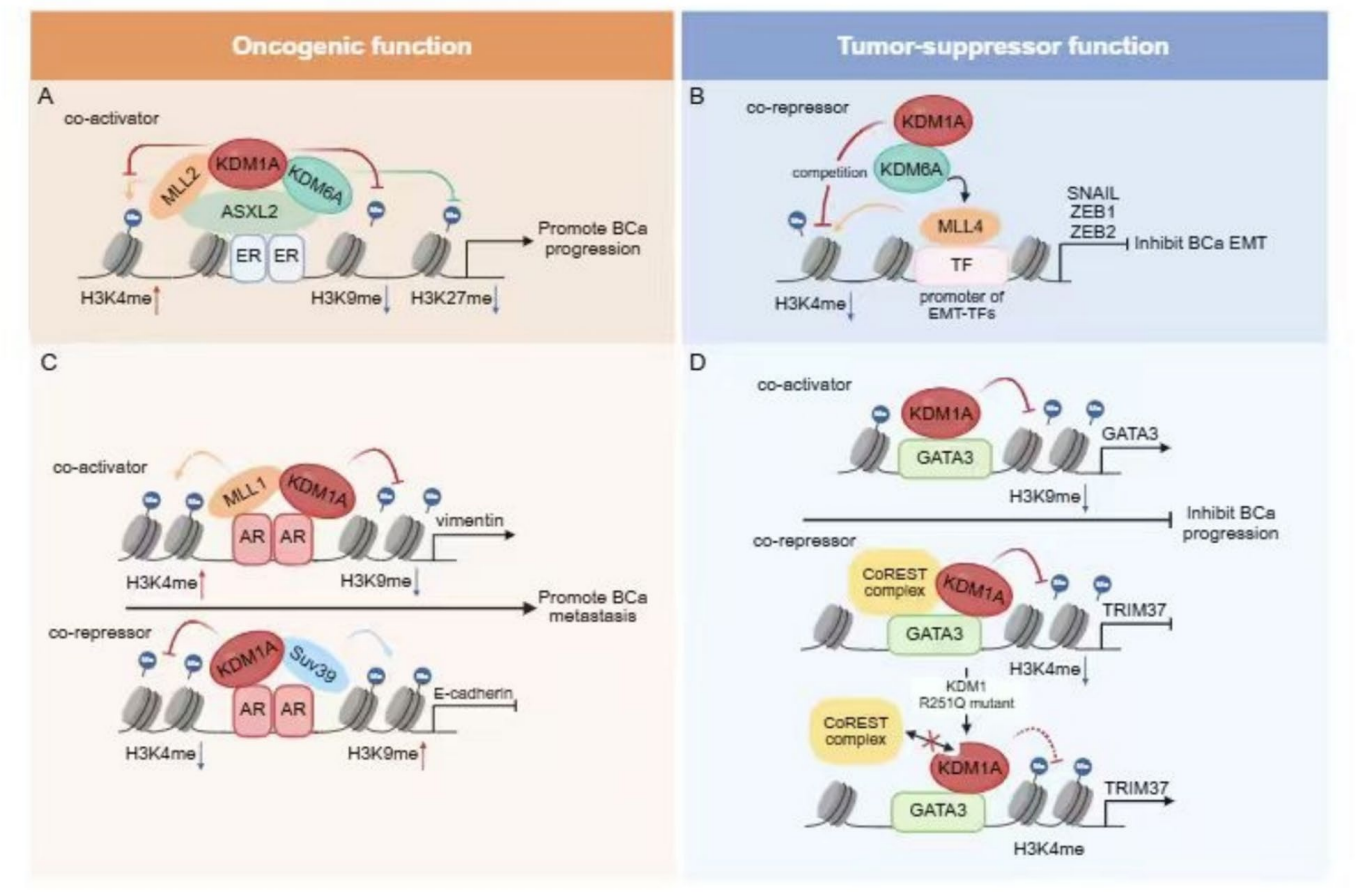
KDM1A possesses a dual role as both a co-activator and a co-repressor of transcription factors to exert multifaceted functions in breast cancer. **A** ASXL2 as an ERα binding protein recruits the KDM1A/KDM6A/MLL2 complex on the promoter of ERα target genes, subsequently KDM1A and KDM6A removing repressive histone codes H3K9me2 and H3K27me2/3 separately. MLL2 rises the active code by increasing H3K4me3 level. Thus, this complex up-regulates ERα action by different histone modification crosstalk to promote breast cancer progression. **B** KDM1/KDM6A/MLL4 complex engages other transcription factors but ERα, the resulting histone-code program is inverted. At the outset, MLL4 occupies the promoters of genes encoding EMT transcription factors (EMT-TFs), and facilitates the expression of EMT-TFs, including SNAIL, ZEB1 and ZEB2. KDM6A blocks the transcriptional activation pattern through inducing the H3K4me2 modification competition between MLL4 and KDM1A. Finally, the KDM1A/KDM6A/MLL4 complex epigenetically inhibits EMT-TFs-mediated transactivation, suppressing EMT-mediated breast cancer stem cell properties. **C** As for the modulation of KDM1A on AR action in luminal breast cancer, the function of KDM1A is conflicting in the epigenetic modification of different AR target genes depending on the specific associated proteins. KDM1A plays an important synergistic role in AR-mediated EMT through contrarily regulating two genes with opposite functions, one is an EMT marker vimentin, the other is E-cadherin, which is negatively correlated with tumor metastasis. KDM1A removes H3K9me2 with a H3K4 methyltransferase (MLL1) on the promoter regions of vimentin to activate gene transcription, while removes H3K4me2 with a H3K9 methyltransferase (Suv39H1) to inhibit E-cadherin transcription, subsequently promoting breast cancer metastasis. **D** The regulatory effect of KDM1A on the same transcription factor (GATA3) can either activate or inactivate the expression of downstream genes to suppress breast cancer progression in the same subtype of breast cancer. KDM1 exerts H3K9 demethylation to enhance the expression of *GATA3* itself on the promoter of *GATA3*. The combined action of both KDM1A and GATA3 promotes the expression of downstream cell adhesion-related genes, thereby hindering tumor metastasis. On the other hand, KDM1A is able to inhibit GATA3-mediated oncogene TRIM37 expression through H3K4 demethylation. The R251Q mutant of KDM1 abolished the interaction between KDM1A and CoREST, enhancing TRIM37 expression

**Fig. 5:**
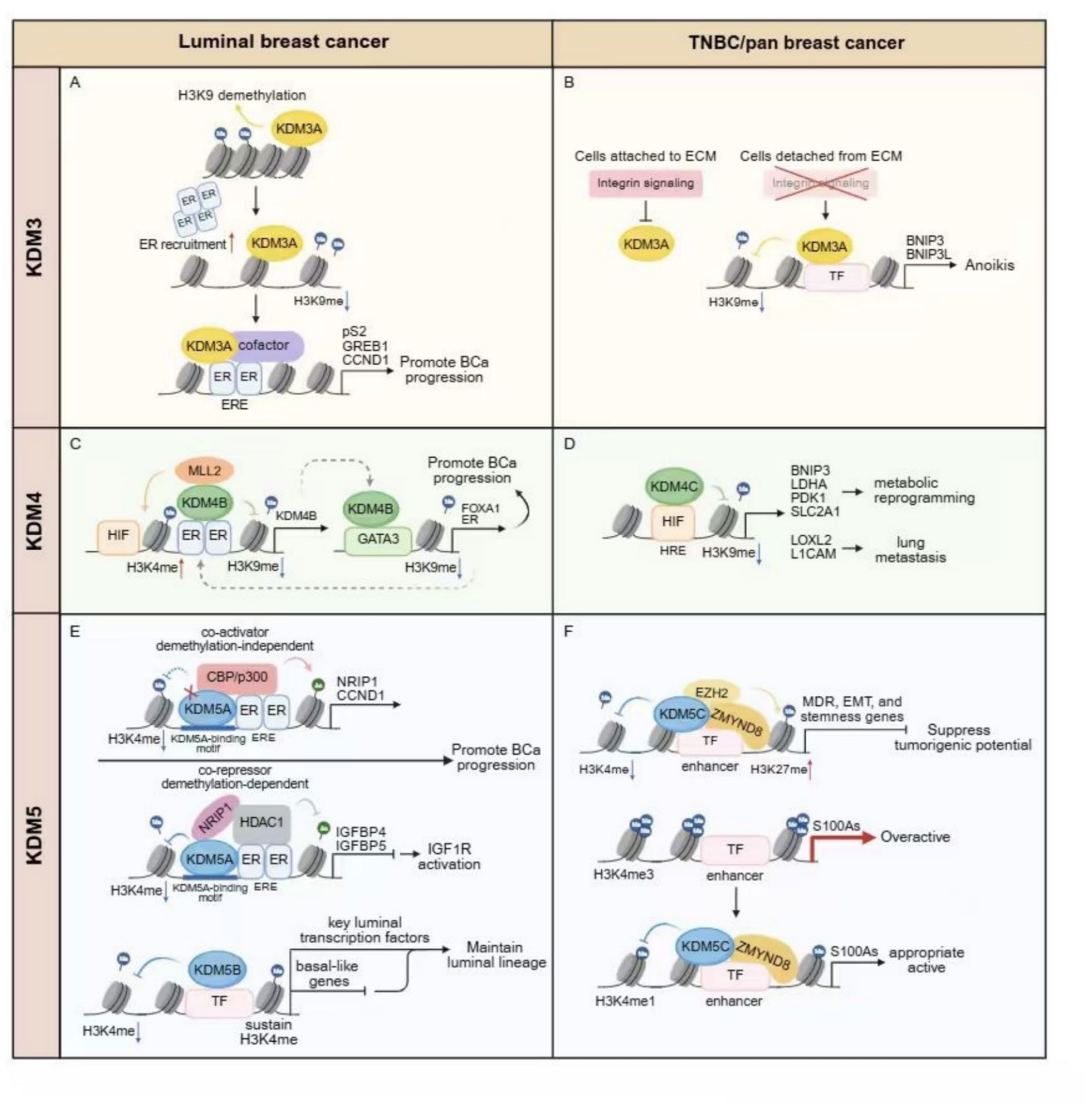
The intricate modulation functions of KDM3/4/5 on multiple transcription factors in breast cancer. **A** KDM3A associates with estrogen receptor-α (ER) and co-activates ER signaling. Upon estrogen stimulation, KDM3A demethylates H3K9me1/2 at estrogen-response elements (EREs) located within promoters and enhancers of downstream genes, thereby facilitating transcription of canonical ER target genes including pS2, GREB1 and CCND1. **B** Beyond its established oncogenic roles, KDM3A can act as a metastasis suppressor. In non-transformed MCF10A mammary epithelial cells, the extracellular matrix (ECM) detachment triggers a rapid KDM3A up-regulation that demethylates H3K9 at the pro-apoptotic loci BNIP3 and BNIP3L, thereby executing anoikis. **C** KDM4B operates with the H3K4 methyltransferase MLL2. Removal of H3K9me3 by KDM4B precedes MLL2-mediated H3K4me3 deposition, establishing a temporally ordered “demethylate-then-methylate” switch that co-activates ER-induced transactivation. KDM4B interacts with transcription factor GATA3 to co-activate GATA3-mediated *ER* and *FOXA1* gene expression. In addition, KDM4B itself carrying functional estrogen-response elements is regulated by ERα, forging a feed-forward loop that amplifies estrogen signaling. **D** KDM4C associates with HIF-1α to enhance HIF-1α-induced transactivation via it demethylase activity. KDM4C is involved in demethylation of histone H3K9me3 at hypoxia-response elements of HIF-1α target genes, thereby driving expression of a series of genes associated with metabolic reprogramming (*BNIP3, LDHA, PDK1, SLC2A1*) and breast cancer lung metastasis (*LOXL2, L1CAM*). **E** KDM5A toggles between transcriptional repression and activation by assembling distinct, context-dependent complexes. Repression requires its demethylase activity, whereas activation occurs independently of catalysis. KDM5A together with ER is recruited to NRIP1 and CCND1 promoters via KDM5A-binding motifs (CCGCCC) in an enzyme-independent manner to assemble transcriptionally active complexes (CBP/p300). Conversely, KDM5A forms a repressive complex with NRIP1 and HDAC1 on IGFBP4/5 promoters, removing H3K4me2/3 on it to inactivate gene transcription. The consequent loss of IGFBP4/5 releases IGF ligands, activates IGF1R signaling, and accelerates luminal breast cancer progression. KDM5B is a critical gatekeeper of luminal lineage identity in breast epithelium. KDM5B represses a panel of basal markers by removing H3K4me, while simultaneously enhancing the expression of key luminal transcription factors (ER, GATA3, FOXA1, TFAP2C) via sustaining H3K4me level, an event that ultimately drives luminal-to-basal lineage infidelity and fuels tumorigenic outgrowth. **F** KDM5C associated with ZMYND8 is recruited to the promoters of target genes, modulating gene transcription. KDM5C interacts with ZMYND8 and EZH2 to decrease H3K4me3 and increase H3K27me3 levels on the promoters of multidrug resistance (MDR), EMT, and stemness genes to suppress chemotherapeutic-induced tumorigenic potential. KDM5C exerts its demethylase activity at enhancers by converting H3K4me3 to H3K4me1, maintaining normal enhancer function. This mechanism serves as a “brake” on enhancer activity, preventing the overexpression of oncogenic genes such as S100A

**Fig. 6:**
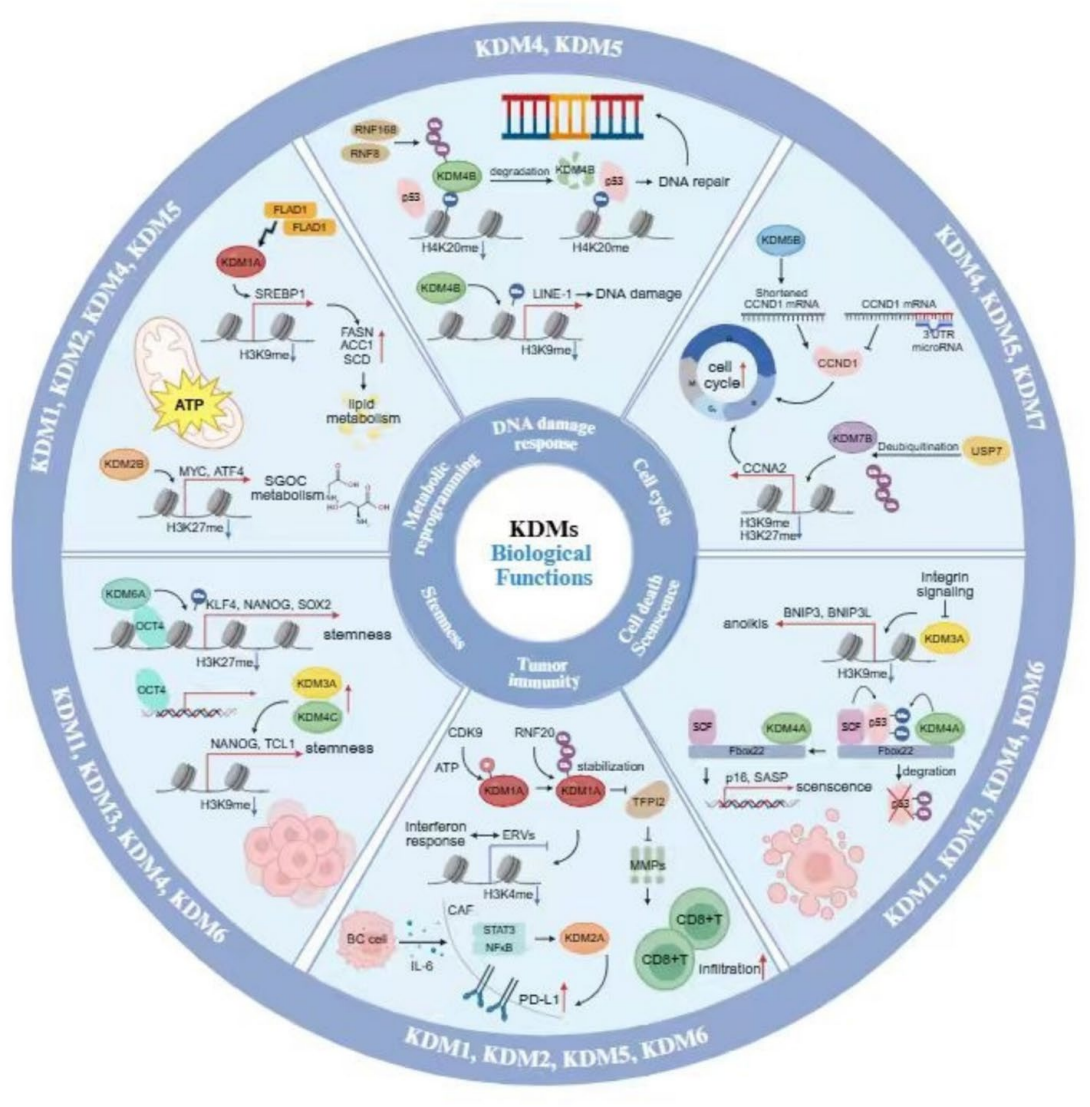
Schematic illustration of biological function of KDM enzymes in breast cancer. The KDM enzymes are involved in demethylation of its specific histone modulation of oncogenic transcription as indicated to underlie a series of biological functions in breast cancer hallmarks

**Fig. 7:**
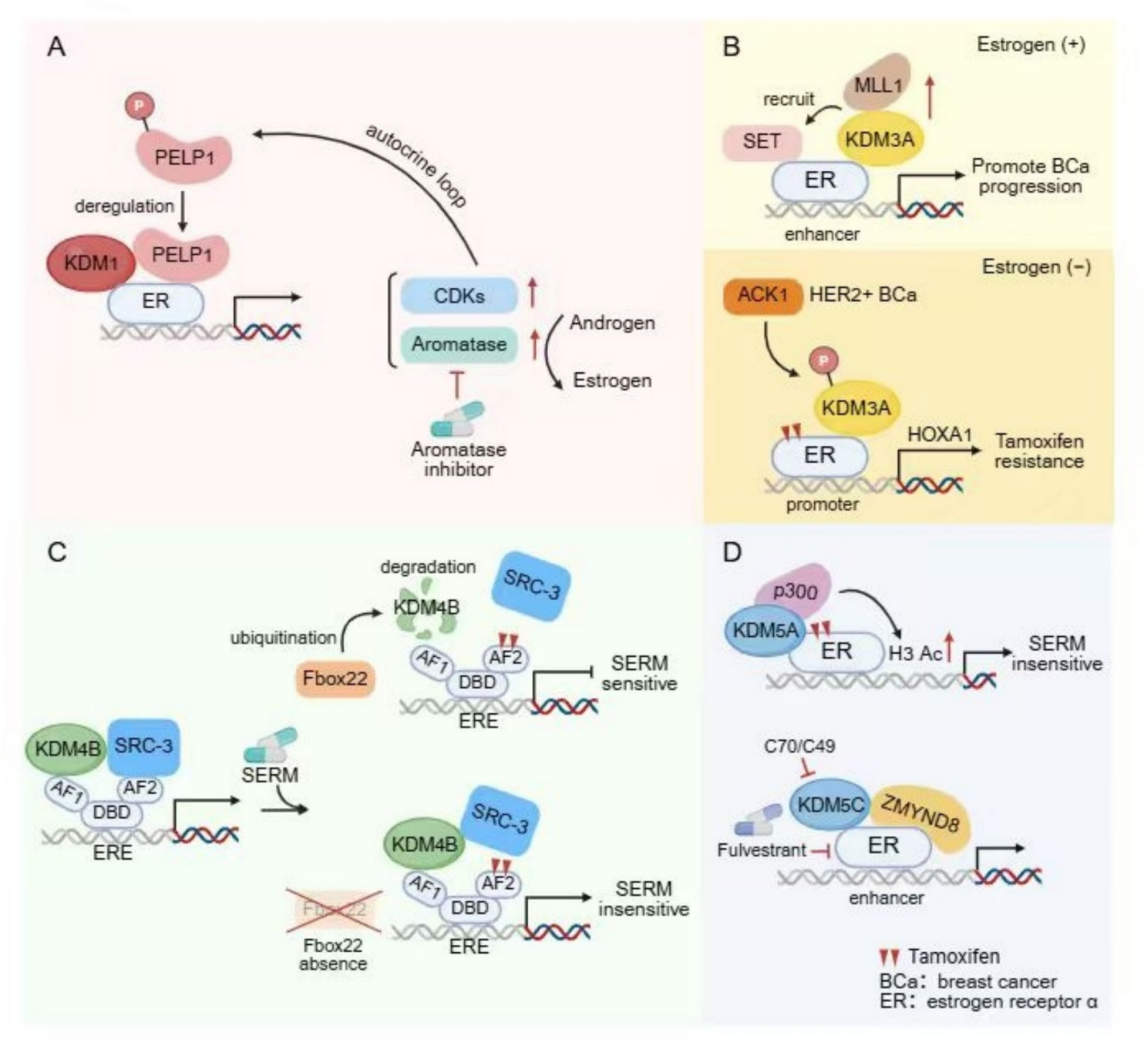
The KDM family proteins participates in abnormal regulation of ER-mediated transactivation to confer endocrine insensitivity/resistance. **A** ER co-regulator PELP1 interacts with KDM1 to upregulate ERα transcriptional activity. PELP1-KDM1 positively regulates the HER2-aromatase pathway, increasing aromatase expression and contributing to aromatase inhibitor (AI) resistance. **B** KDM3A directly associates with ER and co-activates ER action. Upon estrogen stimulation, KDM3A demethylates H3K9me1/2 at estrogen-response elements (EREs) located within promoters and enhancers, thereby facilitating transcription of canonical ER target genes. In estrogen-deprived settings, ACK1 phosphorylates KDM3A, enabling KDM3A to activate the expression of HOXA1, a pro-oncogenic downstream target of ER in the presence of tamoxifen. **C** Fbxo22-mediated KDM4B degradation determines selective estrogen receptor modulator (Tamoxifen) activity in luminal breast cancer. KDM4B acts as a critical ER co-activator. It docks directly onto ER, erases the repressive H3K9me3 mark at estrogen-responsive elements (ERE) and thus licenses ER target-gene transcription. KDM4B binds to the ER AF1 domain and associates with steroid receptor coactivator 3 (SRC-3) to facilitate ER transcriptional activity. During endocrine therapy with Tamoxifen in Tamoxifen sensitive state, KDM4B degradation by Fbox22-mediated unbiquitination releases SRC-3 association with ER, thereby leading to ER action inactive. When Fbox22 is down-regulated in luminal breast cancer, KDM4B/SRC-3 complex co-activates transcriptional activity of ER, resulting in Tamoxifen insensitive. **D** With the treatment of Tamoxifen, KDM5A cooperates with the histone acetyltransferase p300 to enhance the expression of ER target genes, such as NRIP1 and CCND1, consequently leading to endocrine resistance. KDM5C directly interacts with ER and ZMYND8 at ER enhancer regions, thereby promoting the transcriptional activation of ER downstream target genes to promote cell proliferation in breast cancer. The compounds targeting ER and KDM5C exert synergistic therapeutic benefits

KDMs are a family of enzymes are mainly categorized into seven subfamilies (KDM1-7) based on their catalytic mechanisms and substrate specificities (Figs. 1 and 2) [5–7]. The KDM enzymes remove methyl groups from lysine residues on histones and non-histone proteins. KDMs are involved in distinct histone modifications with the assistance of co-regulator complex independent on demethylation activities of KDMs themselves to exert the important functions in cancer progression (Table 1 and Figs. 3, 4, 5). In addition, upstream regulation of KDM expression via multiple post-translational modifications and epigenetic regulation is a rapidly growing area of research in cancer epigenetics, providing the potential strategies for novel drugs for cancer treatment (Table 2). Each KDMs exhibits distinct functions in various biological processes, including cell cycle progression, senescence, therapeutic resistance, tumor metabolism, stemness maintenance, tumor microenvironment to promote tumor proliferation/metastasis and so on (Figs. 6 and 7) [100–102].

KDM1A, also known as lysine-specific demethylase 1 (LSD1), has been shown to act as both a transcriptional activator and repressor by modulating histone H3K4 and H3K9 methylation, thereby influencing estrogen receptor (ER) and androgen receptor (AR) signaling pathways (Table 1 and Figs. 4). KDM2A/KDM2B respectively targeting H3K36 and H3K4 demethylation are involved in cell cycle regulation via transcriptional repression. KDM3A, KDM4, and KDM5 family members further contribute to breast cancer development through their effects on gene expression and chromatin accessibility (Table 1 and Fig. 5). Additionally, KDMs exhibit subtype-specific functions in breast cancer, with KDM1A and KDM5B playing crucial roles in luminal breast cancer by regulating ER-mediated transcription, while KDM4C and KDM6A are more prominently involved in triple-negative breast cancer (TNBC) through mechanisms related to stemness and metastasis (Table 1).

Given the central role of KDMs in breast cancer biology, targeting these enzymes represents a promising therapeutic strategy. Inhibitors targeting KDM1 and KDM4-7 have shown potential in preclinical studies to enhance the efficacy of endocrine therapy, chemotherapy, and targeted therapy by modulating oncogenic signaling pathways (Table 3). Therefore, a comprehensive understanding of the molecular mechanisms and biological functions of KDMs in breast cancer is essential for the development of novel therapeutic approaches.

In this review, we summarize the roles of KDM family members in breast cancer, focusing on their molecular mechanisms, biological functions, and potential as therapeutic targets. We also discuss the challenges and future directions in targeting KDMs for the treatment of breast cancer, highlighting the need for further research to elucidate their complicated mechanisms and provide clinical applications.

## Molecular mechanisms underlying the functions of KDM family enzymes in breast cancer

KDMs are broadly classified into two types, flavin adenine dinucleotide (FAD)-dependent KDM1s and Jumonji C (JmjC) domain family proteins (Figs. 1 and 2). Lysine demethylases (KDMs) erase methyl marks from both histone and non-histone substrates, yet their impact on cancer progression extends far beyond this catalytic reaction. By assembling with co-regulator complexes that operate independently of their demethylase activity, KDMs impose distinct histone signatures that orchestrate oncogenic transcriptional programs (Table 1 and Figs. 3, 4, 5). Concomitantly, an expanding body of work shows that the expression and function of KDMs are themselves tightly governed by multi-layered post-translational modifications and epigenetic inputs, positioning these enzymes at the hub of feed-forward and feedback loops within the cancer epigenetics (Table 2). Elucidating how KDMs integrate catalytic and non-catalytic functions and how their activity is tuned by upstream signaling has become a priority for the development of next-generation, context-specific anti-cancer epigenetic drugs.

**Table 1:** The multifaceted roles of KDM proteins in regulating signaling pathways in breast cancer

KDM proteins	BC subtype	Functions in BC	KDMs predict prognostic	Transcriptional regulation	Interacting proteins	Signaling pathways	Genes	Experimental Model
KDM1	Luminal	promoter	poor	co-activator	PELP1 [8, 9], ASXL2, KDM6A, MLL2 [10]	ER signaling [8, 10, 11]	MAF [11],	both
		suppressor	favorable [12, 13]	co-repressor	Co-REST [12],SIN3A, HDAC [13], NuRD, KDM5B [14]	GATA3 signaling [12]	CCL14 [14], TRIM37 [12], CASP7, TGFB2, CDKN1A(p21), HIF1A, TERT, and MDM2 [13],	both
	TNBC	promoter	poor [15, 16]	co-repressor	CoREST/HDAC [16], HDAC5 [15]	HIF stabilization [17]	FAS, CTDSPL, ISG15, GLIPR1, CYLD, EGLN1, EGLN3, TFPI2, PPP2R1B [16], Cyclin D1, CDK6 [15]	both
	Pan-BC	promoter	not shown	co-activator	MLL1 [18]	AR signaling [18], EZH2 stabilization [19]	NDRG1[20]	both
			poor [18]	co-repressor	SUV39 [18], TBX2/ZNF217 [20]			
		suppressor	favorable (CtBP) [21]	co-repressor	SIX3 [22], ZNF516, CtBP, CoREST [21]	TGF-β signalling [23], Wnt signaling [22], EGFR signaling [21]		both
KDM2A	Luminal	promoter	not shown	co-repressor	NEDD4 [24]	ER/BAER signaling [24]	TET2 [25]	in vitro
		suppressor	not shown	co-repressor			rRNA [26, 27], E2F1 [28]	in vitro
	TNBC	promoter	not shown	co-repressor	RelA [29]		TET2, EpCAM, E-cadherin [29]	in vitro
KDM2B	Luminal	promoter	not shown	co-repressor		Estrogen-related receptor α (ERRα) signaling, proliferator-activated receptor gamma coactivator 1β (PGC1β) signaling [30]		both
	TNBC	promoter	poor [31, 32]	co-repressor			p15INK4B, p16INK4A and p57KIP2 [31], ATF4, MYC [32]	both
	Pan-BC	promoter	poor [33]	co-repressor			ALDH, CD44, CD24 [33]	both
KDM3	Luminal	promoter	poor [34]	co-activator	KDM4B, FOXA1 [35], MLL1, SET [36]	ER signaling [37, 38]	HOXA1 [38]	both
	TNBC	promoter	not shown	co-activator			p53 [39], BNIP3, BNIP3L [40]	both
	Pan-BC	promoter	not shown	co-activator		JAK2-STAT3 signaling [41]		in vitro
KDM4A	Her2 +	promoter	poor [42]	co-activator			GMCSF [42]	both
	TNBC	promoter	not shown	co-repressor	NCoR, HDAC [43]	TRAIL signaling, DR5 signaling [43]		both
	Pan-BC	promoter	not shown	co-repressor	E2Fs, HDACs [44]		ARHI [44]	both
KDM4B	Luminal	promoter	poor [45]	co-activator	MLL2 [46]	ER signaling [46]	CCND1, CCNA1, WEE1 [45]	both
KDM4C	Her2 +	promoter	poor [42]	co-activator			GMCSF [42]	both
	TNBC	promoter	not shown	co-activator		HIF-1α signaling [47]	NOTCH1 [48], BNIP3, LDHA, PDK1, SLC2A1 [47], LOXL2, L1CAM [47]	both
KDM5A	Luminal	promoter	poor [49]	co-activator	p300 [49]	EGFR signaling, HER2 signaling, PI3K-AKT signaling [49]		both
				co-repressor	HDAC1, NRIP1 [49], EMSY, SIN3B, ZNF131 [50],			both
	TNBC	promoter	poor [51, 52]	co-repressor			p16 [51], DDK1 [53], p21, BAK1 [52]	both
KDM5B	Luminal	promoter	poor [54]	co-repressor	HDAC4 [55]	ER signaling, GATA3 signaling, TFAP2C signaling, TGF-β signaling [54],	KRT5, KRT14 [54], p21[56]	both
	TNBC	promoter	not shown	co-activator	EMSY [57]		BRCA1 [58], NANOG, POU5F1, SOX2 [57]	both
		suppressor	not shown	co-repressor	SIN3A, KLF9, HDAC2 [59]		ITGA6, ITGB1 [59]	both
	Pan-BC	promoter	poor [60–62]	co-repressor		AMPK signaling [63], STING signaling [61, 62]	CUX2, SOX17 [64, 65], HEXIM1 [60], FASN, ACLY [63]	both
KDM5C	Luminal	promoter	poor [66]	co-activator	ZMYND8 [66]	ER signaling [66]		both
	Pan-BC	suppressor	not shown	co-repressor	EZH2, ZMYND8 [67]		MCAM [68]	both
KDM6A	Luminal	promoter	not shown	co-activator	CBP, KDM7A [69]	ER signaling [69]		in vitro
		suppressor	favorable [70]	co-activator	MLL4 [70]	GATA3 signaling [70]	DICER [70, 71]	both
	TNBC	promoter	poor [72]	co-activator	SMARCD3, p300 [73], MLL4 [72]	FOXO3 signaling [73]	MMp3 [74], MMP-9, MMP-11, Six1, Cyclin A1, Pim-1, CSPG4, ERBB3 [72]	both
		suppressor	not shown	co-repressor		TGF-β signaling [75]		in vitro
	Pan-BC	promoter	not shown	co-activator	S100A10, ANXA2, PT6 [76]	OCT4 signaling [76, 77]	NANOG, SOX2, KLF4 [76]	both
		suppressor	not shown	co-activator	KDM1, MLL4, HDAC1 [78]		SNAIL, ZEB1, ZEB2 [78]	both
KDM6B	Luminal	promoter	poor [79]	co-activator	BCL-2 [80], IGFBP5 [79]			both
	Her2 +	promoter	not shown	co-activator			tRF-27 [81]	both
	TNBC	promoter	not shown	co-activator			SNAI1 [82]	in vitro
	Pan-BC	promoter	not shown	co-activator		β-catenin signaling, c-Myc signaling [83]		both
		suppressor	not shown	co-activator			IL-6, IL-1β, TNF-α [84]	both
KDM7A	Luminal	promoter	poor [85]	co-activator			RHOJ [85]	both
	Her2 +	promoter	not shown	co-activator			IL-6 [86]	both
	TNBC	promoter	poor [87]	co-activator			BCL2 [88], MKRN1 [87]	both
KDM7B	Luminal	promoter	not shown	co-activator			CCNA2 [89]	both
	TNBC	promoter	not shown	co-activator			SNAI1, ZEB1 [90]	both

### KDMs regulate gene transcription via histone demethylation

#### KDM1

KDM1A (LSD1) demethylates H3K4me1/2 and H3K9me1/2 histone marks through a FAD-dependent enzymatic oxidative mechanism [113]. Its catalytic function of demethylating H3K4me1/2 or H3K9me1/2 is regulated by utilizing FAD, which is different from JmjC-dependent members of KDMs (KDM2-7) [114]. Lysine-specific demethylase 2 (LSD2/KDM1B) as a homolog of LSD1, which specifically demethylases histone H3K4me1/2, exerts an essential role in establishing the DNA methylation imprints during oogenesis [115]. Until now, KDM1B has been confirmed to be associated with cancer cell stemness and is significantly overexpressed in basal-like breast cancer cell lines [116, 117].

KDM1A acting as a crucial histone demethylase has a dual role in gene transcriptional regulation, participating in demethylation of histone H3K4 and H3K9 to regulate gene transcription in opposite directions. KDM1A is identified as an essential component in the different complex. Its diverse regulatory functions also derived from its interaction with distinct co-activators or co-repressors (Figs. 4) [118]. KDM1A inhibits transcriptional activation by histone H3K4 demethylation dependent on its demethylase activity. KDM1A usually functions as a transcriptional repressor in different forms of corepressor complexes, including corepressor for element-1-silencing transcription factor (CoREST) [119], carboxy-terminal binding protein (CtBP) [118], and nucleosome remodeling and deacetylase complex (NuRD) [23]. On the other hand, KDM1A also activates transcription through converts preferably demethylation sites from H3K4 to H3K9 methylation in the modulation of nuclear receptors such as androgen receptor (AR), and estrogen receptors (ERs) [18, 120, 121].

Sexual hormone receptors, including ERα and AR, are essential factors in hormone receptor (HR)-positive breast cancer progression. KDM1A functions as co-activator of hormone receptors and plays an important role in hormone responsive breast cancer. KDM1A participates in co-activation of ERα or AR-mediated gene transcription through demethylation of the H3K9 site [120, 121]. KDM1A is also crucial in the regulation of ERα action via association with other co-regulators [8, 11]. Proline glutamic acid and leucine-rich protein 1 (PELP1) as an ERα co-regulator recruits KDM1 to the ER binding sites and induces reduction of H3K9 methylation. Furthermore, KDM1-specific inhibitor NCL-1 reduces PELP1-induced cell proliferation in MCF-7 cells [8]. It has been shown that amplification of MAF positively correlated with the bone metastasis via redistributing ERα on chromatin to target metastasis-associated genes. During this process, KDM1A as a key epigenetic regulator facilitates the expression of the pro-metastatic MAF/oestrogen-driven gene expression program, and knockdown of KDM1A prevents this metastasis in ER positive breast cancer [11]. Additional sex comb-like 2 (ASXL2) as an ERα binding protein recruits the KDM1A/KDM6A/MLL2 complex on the promoter of ERα target genes, subsequently KDM1A and KDM6A removing repressive histone codes H3K9me2 and H3K27me2/3 separately. Moreover, MLL2 rises the active code by increasing H3K4me3 level. Thus, this complex up-regulates ERα action by different histone modification crosstalk (Fig. 4) [10]. Interestingly, when the KDM1/KDM6A/MLL4 complex engages other transcription factors but ERα, the resulting histone-code program is inverted. At the outset, MLL4 occupies the promoters of genes encoding epithelial–mesenchymal transition (EMT) transcription factors (EMT-TFs), and facilitates the expression of EMT-TFs, including SNAIL, ZEB1 and ZEB2. KDM6A blocks the transcriptional activation pattern through inducing the H3K4me2 modification competition between MLL4 and KDM1A. Finally, the KDM1A/KDM6A/MLL4 complex epigenetically inhibits EMT-TFs-mediated transactivation, suppressing EMT-mediated breast cancer stem cell properties (Fig. 4) [78]. While when KDM1A functions as a component of the MLL1 activator complex, a histone H3K4 methylase, transcriptional activation occurs through KDM1A-catalyzed H3K9 demethylation [118]. As for the modulation function of KDM1A on AR action, KDM1A is involved in the different histone modifications on AR target genes depending on the specific associated proteins. KDM1A plays a synergistic role in AR-mediated EMT relevant gene transcription. KDM1A separately removes H3K9me2 with a H3K4 methyltransferase (MLL1) on the promoter regions of *vimentin*, while removes H3K4me2 with a H3K9 methyltransferase (Suv39H1) on the cis-elements of *E-cadherin* (Fig. 4) [18]. Taken together, KDM1A acts as a crucial co-regulator of transcription factors dependent on its associated protein complexes and chromatin contexts of target genes.

KDM1A associates with the luminal-specific transcription factor GATA3, which is a marker of positive prognosis in breast cancer. The loss of GATA3 is often associated with tumor metastasis [122–124]. KDM1A is capable of inhibiting the expression of the oncogene TRIM37 through H3K4 demethylation in a GATA3-dependent or independent manner. However, on the promoter of *GATA3*, KDM1 performs H3K9 demethylation to enhance the expression of *GATA3* itself. Additionally, the combined action of both KDM1A and GATA3 promotes the expression of downstream cell adhesion-related genes, thereby hindering tumor metastasis in luminal breast cancer (Fig. 4) [12]. KDM1A exerts an anti-tumor effect by interacting with GATA3 and CoREST. Further research revealed that the R251Q mutant of KDM1 almost completely abolished the interaction between KDM1A and CoREST. Disruption of combination between KDM1A and CoREST increases the TRIM37 expression, resulting in a weakened ability of KDM1 to suppress tumor invasion and metastasis (Fig. 4) [12, 125]. Taken together, KDM1A exerts a dual transcriptional function on the same transcription factor.

KDM1A regulates various transcription factors via participating in different transcription complexes in breast cancer. ZNF750 is a zinc-finger transcription factor that associates with tumor suppression and its deregulation can indeed result in neoplastic transformation [126, 127]. ZNF750 recruits KDM1A and HDAC1 to suppress the transcription of LAMB3 and CTNNAL1, inhibiting cell migration and invasion. While additional study suggest the tumor suppressive function of the ZNF750/CTNNAL1-LAMB3 axis is only observed in luminal B breast cancer and TNBC [128]. The tumor-suppressive effect of KDM1A observed in selective breast cancer subtypes appears to depend on whether KDM1A is involved in the regulation of the specific transcription factors. The CoREST/KDM1 complex is recognized for its role in transcriptional repression in breast cancer. Meanwhile, the downstream tumor suppressor or oncogenes co-regulated by CoREST/KDM1 seem to depend on the specific environment of different breast cancer subtypes. Unlike the tumor suppressive role in the luminal subtype [12], the KDM1A/CoREST complex promotes metastasis in TNBC by inhibiting tumor suppressor genes [15, 16]. The tumorigenic role of KDM1 in TNBC has also been mentioned in other studies. KDM1 is upregulated in ER-negative breast cancer, promoting the expression of downstream genes associated with proliferation, such as p21, ERBB2, CCNA2, and METTL14 [129, 130]. KDM1 serves as a prognostic marker associated with unfavorable clinical outcomes in TNBC [131, 132].

The role of KDM1 in the luminal subtype is intricate. While regulating the subtype-specific transcription factor ER, KDM1A promotes ER-mediated transcription through H3K9 demethylation and generally exhibits oncogenic properties. In contrast, in the context of other transcription factor regulations, KDM1 can form a complex with SIN3A/HDAC, exerting tumor-suppressive effects by inhibiting a series of downstream oncogenes, including *TERT*, *MDM2*, *TGFB2*, *p21*, and *HIF1A* [13]. Notably, the same KDM1-associated complexes, such as CoREST, can play completely opposite roles in the development of ER-positive and ER-negative breast cancer due to the selective regulation of different downstream genes [15, 16, 78]. Moreover, for the same target gene *p21*, KDM1 suppresses its transcription via conventional H3K4 demethylation in the luminal subtype [13], whereas KDM1 promotes *p21* expression through selective H3K9 demethylation in TNBC [129]. This indicates that the choice of demethylation sites by KDM1 may be related to the cellular microenvironment of different classifications of breast cancer. The diverse biological functions of KDM1 in pan-breast cancer depend on its regulatory effects on different transcription factors. The role of KDM1 in pan-breast cancer remains complicated with contradictory effects, exhibiting both oncogenic and tumor-suppressive roles in pan-breast cancer. In the MCF-7 and MDA-MB-231 cell lines, the transcription factor SIX3 recruits the KDM1/NuRD complex to inhibit the oncogenes *WNT1* and *FOXC2*, thereby exerting tumor-suppressive effects [22]. Additionally, studies in MCF7 and MDA-MB-361 have found that TBX2 interacts with ZNF217 and KDM1/CoREST to inhibit the expression of *NDRG1*, which is a negative regulator of cell proliferation, thereby inducing oncogenic activity [20].

#### KDM2

The histone lysine demethylase 2 (KDM2), which are α-ketoglutarate and Fe (II)-dependent dioxygenases, were the first demethylase identified to contain the Jumonji C domain (JmjC) [133]. The KDM2A family consists of two members KDM2A and KDM2B. These two proteins have similar structures which contain the JmjC, zinc-fnger CXXC (ZF-CXXC), plant homeodomain (PHD), F-box, and leucine-rich repeat (LRR) domains (Fig. 1) [114]. These two homologous histone demethylases exhibit different preferences for histone modification sites. KDM2A (also known as FBXL11 and JHDM1A) is capable of demethylating di- and mono-methylated H3K36 (H3K36me1/2), exerting a transcriptional repression effect by erasing the histone transcription-promoting marks [133]. KDM2B (also known as FBXL10 and JHDM1B) identifies and removes histone methylation marks, specifically H3K4me3 [134, 135], H3K36me2 [134, 136], and H3K79me2/3 [137]. Since the aforementioned three histone methylation modification sites are all signs of transcriptional activation, KDM2B is capable of mediating transcriptional repression by demethylating these activating marks through its enzymatic activity, similar to KDM2A (Fig. 2).

KDM2 interacts with the transcription factors and functions as an epigenetic regulatory factor, thereby playing a crucial role in the progression of tumor cells. E2F1 acts as a transcription factor and a tumor suppressor in diverse cellular physiological processes, including inducing cell cycle arrest and promoting apoptosis [138–140]. In breast cancer, KDM2A inhibits the transcription of E2F1-targeted downstream genes. KDM2A has been shown to inhibit the oncogenic matrix metalloproteinase (MMP) family genes in MCF-7 cells and suppress the E2F1 downstream genes *FLT*/*KDR* in MDA-MB-231 cells to promote the cell invasion, metastasis, and angiogenesis [28].

DNA methylation was regarded as a highly stable epigenetic marker until the discovery of the ten-eleven translocation (TET) gene family, which contains TET1, TET2, and TET3. As a common epigenetic modification, the dynamic balance between DNA methylation and demethylation maintains the stability of gene transcription and expression [141, 142]. KDM2A collaborates with RelA to co-occupy at the promoter of TET2 gene, decreasing histone H3K36 methylation level on it to repress the transcription of TET2 and its downstream tumor suppressor genes such as epithelial cell adhesion molecule (EpCAM) and E-cadherin. On the other hand, KDM2A indirectly promotes DNA methylation, thereby advancing tumor progression and enhancing the invasion and metastasis of TNBC [29]. The interplay between KDM2A with ERα inhibits the expression of TET2 through its H3K36 demethylation activity in the luminal subtype breast cancer with significant effect of regulating endocrine disruptors such as bisphenol A (BPA) and S (BPS) induced cell proliferation [25].

KDM2B promotes the proliferation of TNBC cells by transcriptionally repressing the cyclin-dependent kinase inhibitors p15INK4B, p16INK4A, and p57KIP2. Chromatin immunoprecipitation (ChIP) assays demonstrate that KDM2B is recruited to the promoters of these loci, catalyzing the demethylation of histone H3K4me3 and H3K36me2 and consequently attenuating their transcription [31].

Ribosome biogenesis is gated by rRNA transcription, and its dysregulation is increasingly recognized as a driver of tumorigenesis, prompting exploration of rRNA-targeted anti-cancer strategies [143–145]. KDM2A antagonizes this process by demethylating H3K36me2 at ribosomal DNA promoters, thereby repressing rRNA transcription and curtailing cell proliferation in breast cancer [26, 27, 146]. Expression of KDM2A can be pharmacologically or nutritionally induced by antioxidants (gallic acid, propyl gallate, EGCG) [27], or by energy–stress mimics such as metformin or mild glucose restriction [26]or under mild glucose starvation [146]. Polycomb group (PcG) proteins play a crucial role in epigenetically silencing gene transcription by modifying histones, specifically through the tri-methylation of histone H3 at lysine 27 (H3K27me3) and the ubiquitination of histone H2A at lysine 119 (H2AK119ub) [147]. Beyond ribosomal control, KDM2 family proteins interface with Polycomb-mediated gene silencing. KDM2A/B incorporate into non-canonical polycomb repressive complex (PRC) 1.1 complexes via scaffolding interactions with PCGF1, RING1B, RYBP, SKP1 and BCOR [148–150]. KDM2B additionally couples its H3K36 demethylase activity to PRC2 recruitment, silencing PRC-targeted microRNAs [33]. Collectively, KDM2 enzymes operate as versatile chromatin modulators, repressing both protein-coding mRNAs and non-coding RNAs-including rRNA and miRNA—to orchestrate global transcriptional output.

#### KDM3

The KDM3 subfamily, comprising KDM3A, KDM3B, and KDM3C, selectively demethylates mono- and di-methylated H3K9 to license transcriptional activation [151]. Because KDM3A is the best-characterized member, the following discussion centers on its regulatory functions.

KDM3A is frequently overexpressed across multiple malignancies and functions as a bona fide oncogene. By erasing repressive H3K9me1/2 marks, it potentiates transcription-factor activity and installs pro-tumorigenic transcriptional programs that accelerate cancer progression [34, 152, 153]. KDM3A contains a conserved LXXLL motif that mediates direct interaction with nuclear receptors. Deletion or mutation of this motif abolishes its co-activator function in androgen receptor (AR)-dependent transcription (Fig. 1) [151]. The KDM3A also directly associates with estrogen receptor-α (ER) and co-activates ER signaling. Upon estrogen stimulation, KDM3A demethylates H3K9me1/2 at estrogen-response elements (EREs) located within promoters and enhancers, thereby facilitating transcription of canonical ER target genes including pS2, GREB1 and CCND1 (Fig. 5) [36, 37]. In estrogen-deprived settings, tyrosine kinase ACK1 phosphorylates KDM3A to sustain expression of HOXA1 (Fig. 7) [38]. Recruitment is further reinforced by the histone-binding oncoprotein SET, which co-occupies EREs with ER and nucleates a co-activator complex comprising KDM3A and MLL1 (Fig. 7) [36]. Conversely, RNAi-mediated KDM3A depletion increases H3K9me1/2 and compromises ER chromatin occupancy. The cooperative recruitment of KDM3A by ER itself and its binding proteins reinforces the close connection between KDM3A and ER. Furthermore, depletion of KDM3A by RNAi abrogates the recruitment of ER to the cis-regulatory elements via modulating the methylation of H3K9me1/2, illustrating bidirectional cooperativity between KDM3A and ER [37]. Collectively, KDM3A-ER interplay, underpinned by H3K9 demethylation, is indispensable for the transcriptional circuitry that drives luminal breast cancer.

KDM3A also serves as a co-activator for non-steroid transcription factors. It associates with STAT3 at the MYC promoter, and IL-6-triggered, JAK2-dependent phosphorylation of KDM3A potentiates its H3K9me2 demethylase activity to amplify STAT3 signaling and oncogenesis [41]. Beyond ER-positive breast cancer, KDM3A fuels triple-negative breast cancer (TNBC) proliferation and invasion. The transcription factors zinc-fingers and homeoboxes 2 (ZHX2) and hypoxia-inducible factor 1-alpha (HIF-1α) co-occupy active chromatin regions (H3K4me3/H3K27ac) that drive KDM3A and downstream targets AP2B1, COX20, and PTGES3L. Depletion of ZHX2 reduces KDM3A expression and halts proliferation, whereas enforced KDM3A expression rescues the growth defect, underscoring its essential role in TNBC [154].

Beyond its established oncogenic roles, KDM3A can act as a metastasis suppressor. In non-transformed MCF10A mammary epithelial cells, the extracellular matrix (ECM) detachment triggers a rapid KDM3A up-regulation that demethylates H3K9 at the pro-apoptotic loci BNIP3 and BNIP3L, thereby executing anoikis. This response is selectively lost in anoikis-resistant breast cancer cells (Fig. 5) [155]. Concordantly, CRISPR or RNAi-mediated KDM3A depletion in the weakly metastatic mouse lines 67NR and 4T07 markedly increases lung colonization, demonstrating that KDM3A-mediated anoikis constrains dissemination of breast cancer [40]. Thus, KDM3A exerts context-dependent, even opposing, functions in mammary tumorigenesis. It sustains ER-positive tumor growth through hormone-dependent and -independent transcriptional programs, yet simultaneously safeguards against metastatic spread by enforcing anoikis. These paradoxical activities underscore the need for further mechanistic dissection before KDM3A can be exploited as either a drug target or a biomarker in anti-endocrine-resistance strategies.

#### KDM4

KDM4 constitutes the largest KDM subfamily and comprises KDM4A-D, which demethylate di- and tri-methylated H3K9/H3K36 [156–158], and tri-methylated H1.4K26 [159]. Whereas H3K9me3 and H1K26me3 mark transcriptionally silent or heterochromatic regions, H3K36 methylation is broadly associated with active transcription; hence KDM4-mediated demethylation can either activate or repress gene expression[160, 161]. KDM4A-C are ubiquitously expressed in human and mouse tissues, whereas KDM4D is testis-restricted [162]. Converging data position KDM4 enzymes as actionable oncogenes whose amplification or overexpression spans multiple tumor types [48, 163–166]. Specifically, KDM4 family members exhibit selective oncogenic effects across breast cancer subtypes [167].

KDM4A is the best-characterized histone demethylase. Granulocyte–macrophage colony-stimulating factor (GM-CSF) functions as an autocrine signaling molecule that activates downstream signaling pathways through its receptor, GM-CSFR and involves in the regulation of the tumor immune microenvironment and can promote tumor cell proliferation [168, 169]. In HER2-positive leptomeningeal breast cancer, KDM4A/C erase H3K9me3 and H3K36me3 at the GM-CSF promoter, generating an open chromatin state that permits GM-CSF transcription [42]. This autocrine cytokine fuels tumor growth by engaging GM-CSFR and remodeling the immune microenvironment. Concomitantly, KDM4A/C-catalyzed demethylation enhances NF-κB occupancy at the GM-CSF promoter, further reinforcing gene activation [42, 170, 171].

Qualitative and kinetic profiling indicates that KDM4A demethylates H3K9me3 approximately fivefold more efficiently than H3K36me3, implying a primary role in transcriptional activation [167]. Nevertheless, functional outcomes are modulated by extensive cross-talk among H3K9, H3K36 and H1.4K26 modifications and the broader epigenetic landscape [172]. KDM4A also operates as a transcriptional repressor by nucleating a KDM4A-NCoR-HDAC complex. In MDA-MB-231 cells, KDM4A-catalyzed removal of H3K9me3/H3K36me3 is coupled with HDAC-mediated deacetylation of H3K9/H3K27 at the TRAIL and DR5 promoters, silencing these death-receptor genes and blunting TRAIL-based therapy [43]. A similar mechanism targets the imprinted tumor-suppressor ARH1 (DIRAS3), whose expression is normally high in breast and ovarian epithelium but frequently lost in tumors [173, 174]. KDM4A, HDACs and E2F1 co-occupy the ARH1 promoter, repressing transcription; conversely, restoration of ARH1 inhibits KDM4A-driven motility and invasion in breast cancer cells [44]. SiRNA against KDM4A curtails cell growth in TNBC models. Additionally, in the luminal subtype, KDM4A co-activates ER-mediated transcription to sustain tumorigenic growth. Pharmacologic or genetic inactivation targeting the catalytic center of KDM4A might be useful in breast cancer adjuvant therapy [43, 175, 176].

KDM4B is over-expressed in hormone-driven cancers, including breast, prostate and ovarian tumors. KDM4B acts as a coactivator of AR and ERα. Given the crucial roles of AR and ERα in prostate and breast carcinogenesis, KDM4B is considered to be potential drug targets for intervening in these malignancies [177, 178]. KDM4B operates with the H3K4 methyltransferase MLL2. Removal of H3K9me3 by KDM4B precedes MLL2-mediated H3K4me3 deposition, establishing a temporally ordered “demethylate-then-methylate” switch that co-activates ER-induced transactivation. Moreover, KDM4B interacts with transcription factor GATA3 to co-activate GATA3-mediated *ER* and *FOXA1* gene expression. Interestingly, KDM4B itself carrying functional estrogen-response elements is regulated by ERα and hypoxia, forging a feed-forward loop that amplifies estrogen signaling (Fig. 5) [45, 46, 179]. This sequential crosstalk highlights the dynamic choreography underlying hormone- and hypoxia-responsive gene programs. KDM4B is indispensable for luminal tumors (MCF7/T47D). Knockdown of KDM4B in ER-positive MCF7 or T47D cells diminishes cell proliferation and tumor formation in nude mice, whereas no such changes have been observed in ER-negative MDA-MB-231 cells [180].

KDM4C originally identified as gene amplified in squamous-cell carcinoma (GASC1) is recurrently amplified in multiple tumors [165, 181–183]. Functional studies indicate KDM4C acts as a bona fide oncogene. KDM4C depletion curtails cell proliferation and metastasis in breast cancer [47, 48]. KDM4C associates with HIF-1α to enhance HIF-1α-induced transactivation via it demethylase activity. KDM4C is involved in demethylation of histone H3K9me3 at hypoxia-response elements of HIF-1α target genes, thereby driving expression of a series of genes associated with metabolic reprogramming (*BNIP3, LDHA, PDK1, SLC2A1*) and breast cancer lung metastasis (*LOXL2, L1CAM*) (Fig. 5) [47]. In parallel, KDM4C-mediated removal of H3K9me3 at the *NOTCH1* locus endows basal-like cells with stem-like self-renewal capacity. KDM4C preferentially drives transformation of ER-negative lesions Thus, KDM4C is emerging as a biomarker and therapeutic target in TNBC [48].

Taken together, to refine the application of KDM4-directed therapy across the spectrum of breast-cancer subtypes, it may prove beneficial to first stratify patients on the basis of their molecular portraits.

#### KDM5

KDM5A-D (JARID1A-D) constitute the KDM5 sub-family of JmjC demethylases that remove activating H3K4me2/3 marks and, more recently, H3K4me1, thereby enforcing transcriptional repression [184–187]. All four paralogs harbor a catalytic JmjC domain flanked by multiple plant homeodomain (PHD) fingers, three in KDM5A/B and two in KDM5C/D, endowing them with the unique capacity to recognize H3K4 methylation and DNA methylation [54, 114]. In TNBC, PHD1-mediated binding to H3K4me0 is indispensable for tumor progression [188]. Whereas PHD3 engagement with H3K4me3 appears to fine-tune, rather than fully activate, KDM5B catalytic output [54]. How these PHD-dependent recruitment events intersect with JmjC-mediated demethylation to sculpt gene expression programs remains an open question.

KDM5A was originally identified as retinoblastoma binding protein 2 (RBP2) [189], Since then, it has emerged as a pivotal driver of tumorigenesis and progression across multiple cancer types, and its functions and mechanisms have been most intensively interrogated in breast cancer [190, 191]. In luminal breast cancer, KDM5A toggles between transcriptional repression and activation by assembling distinct, context-dependent complexes. Repression requires its demethylase activity, whereas activation occurs independently of catalysis (Fig. 5) [49]. Insulin-like growth factor binding proteins (IGFBPs) inhibit insulin-like growth factor-1 receptor (IGF1R) activation by blocking ligand-receptor interactions [192]. KDM5A together with ER is recruited to NRIP1 and CCND1 promoters via KDM5A-binding motifs (CCGCCC) in an enzyme-independent manner to assemble transcriptionally active complexes (CBP/p300). Conversely, KDM5A forms a repressive complex with nuclear receptor-interacting protein 1 (NRIP1) and histone deacetylase 1 (HDAC1) on insulin-like growth factor-binding protein 4/5 (IGFBP4/5) promoter, removing H3K4me2/3 on it to inactivate gene transcription. The consequent loss of IGFBP4/5 releases IGF ligands, activates IGF1R signaling, and accelerates ER-positive tumor progression (Fig. 5) [49]. In TNBC, KDM5A represses the expression of downstream tumor suppressor genes in a catalysis-dependent manner. KDM5A removes H3K4me2/3 from the promoter of *p16* and *DKK1*, a negative regulator of Wnt signaling activation, thereby repressing their expression [51, 53]. Beyond repressing tumor suppressor genes, KDM5A also governs a broader oncogenic transcriptome. Paradoxically, it suppresses the expression of PI3K/AKT pathway-related genes such as AKT2 and mTOR, thereby restraining the progression of TNBC [193]. Collectively, these findings highlight suppression and promotion role of KDM5A in breast cancer development. Additionally, studies have shown that the demethylase activity of KDM5A does not directly lead to gene silencing but dynamically regulate gene activation. KDM5A is a core component of the EMSY complex, which is recruited to ZNF131 and co-localizes with H3K4me3-marked active promoter regions to mediate downstream gene activation. The presence of KDM5A in this complex may help maintain transcriptional cyclic waves that are thought to involve methylation followed by demethylation [50].

KDM5B initially named as a novel human gene (*PLU-1*) contains three PHD/LAP motifs, showing consistently expressed in breast cancers. The expression of KDM5B is regulated by signaling from cErbB2 in breast cancer cells [194]. Subsequent study has established KDM5B as a key epigenetic driver in mammary biology. During normal development, its H3K4me3 demethylase activity orchestrates lineage-specific transcription by positively regulating the expression of key developmental factors such as FOXA1, STAT5A, PRLR and FGFR2 [195]. KDM5B adopts a tumor-promoting role in neoplastic contexts. It is recruited to the promoters of tumor-suppressive genes (e.g., *BRCA1*, *CAV1* and *HOXA5*) and erases activating H3K4 methylation marks, thereby inhibiting their expression. This repression compromises DNA-damage repair and accelerates breast cancer progression [58].

Hexamethylene bis-acetamide (HMBA)-inducible protein 1 (HEXIM1) acting as a tumor suppressor is downregulated in multiple cancers [196]. Molecular docking studies indicate that HMBA and 4a1 occupy the methylated lysine pocket of KDM5B substrate, sterically hindering its catalytic removal of H3K4me2/3 marks. HMBA and 4a1 act as the inducers of HEXIM1 to enhance HEXIM1 expression by blocking KDM5B-dependent demethylation on HEXIM1 promoter, providing the novel avenues for identifying KDM5B-targeting compounds for breast cancer therapy [60]. KDM5B similarly suppresses miRNA formation by targeting their promoter regions through its demethylase activity, thus disrupting transcriptional regulatory networks. In particular, KDM5B inhibits miRNA let-7e expression in a demethylase-dependent manner to increase cyclin D1 expression, promoting cell cycle progression in MCF-7 cells [197]. Additionally, KDM5B and ETS-1 jointly recruit EMSY to the promoter of anti-metastatic microRNA miR-31, thereby reducing its expression to promote the invasion/migration in breast cancer cells [198].

Notably, KDM5B regulates gene transcription through its demethylase activity to exert a crucial oncogenic role in gene transcription and increases transcriptional heterogeneity [52, 54]. KDM5B is a critical gatekeeper of luminal lineage identity in breast epithelium. In MCF-7 cells, depletion of KDM5B de-represses a panel of basal markers, while simultaneously eroding the expression of key luminal transcription factors (ER, GATA3, FOXA1, TFAP2C), an event that ultimately drives luminal-to-basal lineage infidelity and fuels tumorigenic outgrowth. Mechanistically, KDM5B is preferentially recruited to highly transcribed promoters marked by dense H3K4 methylation. Rather than extinguishing transcription, its catalytic activity modulates gene expression by locally dampening H3K4 methylation levels, ensuring the balanced expression of lineage-specifying genes that underpins luminal breast cancer initiation and progression (Fig. 5). This paradoxical regulation actually reflects the complex nature of the modulation function of KDM5B on the downstream gene expression. In addition, CCCTC-binding factor (CTCF) depletion diminished KDM5B recruitment to chromatin, revealing that CTCF recruits KDM5B to sculpt the luminal epigenome [54]. Recent studies indicate that KDM5B participates in influencing intratumoral heterogeneity by regulating the transcription of a series of specific genes. This effect promotes phenotypic diversity of tumor cells and confers to treatment resistance [52].

Nevertheless, evidence has demonstrated that KDM5B expression is significantly lower in TNBC relative to ER positive breast cancer, ectopic expression of KDM5B in the MDA-MB-231 cells suppresses cell migration and invasion. KDM5B associates with NuRD complex and cooperates with HDAC1 to gene repression. These results highlight tumor suppression function of KDM5B in TNBC [188]. KDM5B also suppresses cell invasion and metastasis by cooperating with the SIN3A/KLF9/HDAC2 complex to repress the expression of integrin α6 (ITGA6) and integrin β1 (ITGB1) in TNBC [59]. Taken together, KDM5B exerts the diverse roles of across various breast cancer subtypes.

Interestingly, during H3K4 demethylation, KDM1A and KDM5B exhibit temporal specificity based on their distinct methylation state preferences, KDM5B first recognizes H3K4me3, followed by KDM1-mediated demethylation of H3K4me2/1 in both MCF-7 cells and MDA-MB-231 cells. Thus, this collaborative interaction demonstrates the sequential and precise nature of the demethylation process [14].

KDM5C (JARID1C or SMCX) located on the X chromosome was considered a tumor suppressor by regulating enhancer function in breast cancer [199, 200]. Genome-wide histone modification profiling has identified H3K4me1 and H3K27ac as key signatures of enhancers, where active enhancers are typically characterized by the co-localization of these two marks, whereas enhancers marked only by H3K4me1 are often transcriptionally inactive [201]. Recent studies have revealed that H3K4me3 is closely associated with hyperactivation of enhancers, a state that can drive the development of breast cancer and other malignancies [202–204]. KDM5C exerts its demethylase activity at enhancers by converting H3K4me3 to H3K4me1, maintaining normal enhancer function. This mechanism serves as a “brake” on enhancer activity, preventing the overexpression of oncogenic genes such as *MCAM* and *S100A* [68, 202].

Zinc finger MYND-type containing 8 (ZMYND8) known as RACK7 was initially identified as an activator of protein kinase C [205]. ZMYND8 carrying with bromodomain recognizes the histone modification H4K16ac to be involved in regulating gene transcription [206]. ZMYND8 interacts with multiple protein complexes, participating in the formation of enhancer-promoter loops to modulate transcription factor activity [207]. RACK7 recruits KDM5C to enhancer sites, reducing histone H3K4me3 levels and repressing the transcription of several oncogenes, including *S100A2*, *S100A4*, and *S100A6*. Loss of RACK7 or KDM5C results in enhancement of H3K4me3 and H3K27ac level on enhancer sites and increasing eRNAs transcription (Fig. 5) [202]. KDM5C associated with ZMYND8 is also recruited to the promoters of target genes, modulating gene transcription. KDM5C interacts with ZMYND8 and EZH2 to decrease H3K4me3 and increase H3K27me3 levels on the promoters of multidrug resistance (MDR), EMT, and stemness genes to suppress chemotherapeutic-induced tumorigenic potential (Fig. 5) [67]. In luminal breast cancer, the JmjC domain of KDM5C binds with ERα activation function 2 (ERα AF2), subsequently inhibiting its demethylase activity. This inhibition of KDM5C leads to enhancer activation of ERα regulated genes, thereby promoting breast cancer progression [66].

KDM5D (JARID1D or SMCY), a male-specific protein, inhibits the invasion-associated genes *MMP1-3*, *MMP7*, and *Slug* by demethylating H3K4me3 at their promoters, suppressing the invasion of prostate cancer cells. KDM5D as a potential mediator of docetaxel sensitivity in metastatic hormone-sensitive prostate cancer. KDM5D physically associates with AR, and regulates its transactivation by demethylating H3K4me3. Depletion of KDM5D dysregulates AR signaling, resulting in docetaxel insensitivity. Moreover, KDM5D also interacts with p38α, inducing demethylation at K165 and inhibiting p38α phosphorylation [208–210]. While the function of KDM5D in breast cancer is still elusive.

#### KDM6

The KDM6 family comprising KDM6A (UTX), KDM6B (JMJD3), and KDM6C (UTY) is an important group of H3K27me2/3 demethylases that remove repressive histone marks to promote chromatin relaxation and activate specific promoters or enhancers [211, 212]. Since UTY (KDM6C) is male-specific, studies on breast cancer have focused mainly on KDM6A and KDM6B [213]. KDM6 activity is context-dependently regulated by genotoxic stress (such as DNA damage) and hypoxia [71, 214]. In addition to the core enzymatic JmjC domain, KDM6A contains 6 highly conserved tetratricopeptide repeat (TPR) domains that are crucial for protein interactions and essential for demethylase-independent transcriptional regulation (Fig. 1). However, KDM6B does not contain any structural domains other than the JmjC catalytic domain [211]. The KDM6 family is crucial for epigenetic regulation, with their enzyme-dependent or -independent activity. KDM6A is identified to be mutated in a series of cancers [215–217]. Thus, a well understanding the roles of KDM6 family proteins in the physiological and pathological contexts is essential to advance the clinical application of KDM6 modulators.

MLL3 (KMT2C) and MLL4 (KMT2D) are core components of the COMPASS complex and control H3K4me1 at enhancers, which is essential for gene expression regulation to be potential therapeutic targets [218–220]. KDM6A physically associates with MLL3- and MLL4-containing complexes through its TPR domains [221]. KDM6A and MLL4 associate with GATA3, co-activating the expression of its downstream gene *Dicer* in breast cancer [70]. KDM6A and MLL4 drive the expression of a group of oncogenes and pro-metastatic genes, including *MMP-9*, *MMP-11*, and *Six1* in breast cancer cells. KDM6A -catalyzed demethylation of H3K27me3 and MLL4-induced H3K4me3 occurred interdependently at co-regulated genes. KDM6A depletion increased H3K27me3 levels at the proximal promoters of these genes. In addition, high levels of UTX or MLL4 were cooperated with poor prognosis in breast cancer patients. Coordinated regulation of gene expression programs by UTX and MLL4 is coupled to the proliferation and invasion of breast cancer cells [72]. MLL3 and MLL4 as histone H3 lysine 4 methyltransferases are the most commonly mutated genes in triple-negative breast cancer (TNBC). Loss of MLL3/MLL4 enhances the chromatin binding and enzymatic activity of KDM6A, leading to upregulation of the pro-metastatic gene MMP-3. The findings highlight the KDM6A/MMP-3 axis as a key mediator of MLL3/MLL4 loss-driven metastasis in TNBC [74]. Collectively, KDM6A and MLL3/MLL4 not only synergistically co-activate gene expression, but also maintain the balance of histone methylation and transcriptional activity.

It has been reported that KDM6A is a downstream gene of ER or GATA3 [69, 70]. KDM6A can form a positive feedback loop with ER signaling pathway, and is crucial for transcriptional processes in luminal subtype of breast cancer. In ER signaling, KDM6A collaborates with KDM7A to remove H3K27 methylation, followed by CBP-mediated H3K27 acetylation. This methylation-acetylation conversion enhances expression of CXCR4 and KDM6A, thereby activating downstream pathways that drive tumor progression [69]. On the other hand, KDM6A acts as a tumor suppressor in the GATA3 pathway, suppressing tumor metastasis by promoting Dicer expression. Transactivated by GATA3 and cooperating with MLL4, KDM6A dynamically regulates histone methylation to enhance Dicer expression, which exerts tumor-suppressive functions via miRNA maturation regulation [70]. Overall, KDM6A can amplify its effects through positive feedback regulation in luminal breast cancer. However, whether KDM6A inhibits or facilitates cancer development depends on its interactions with specific transcription factors and their targeted genes.

KDM6A plays a critical role in chemotherapy-induced enrichment of cancer stem cells. Paclitaxel induces HIF-1-dependent expression of S100A10, which forms a complex with ANXA2 that interacts with histone chaperone SPT6 and histone demethylase KDM6A. S100A10, ANXA2, SPT6, and KDM6A are recruited to OCT4 binding sites and KDM6A erases H3K27me3 chromatin marks, facilitating transcription of genes encoding the pluripotency factors NANOG, SOX2, and KLF4, which along with OCT4 are responsible for BCSC specification [76]. KDM6A promotes the expression of key pluripotency factors KLF4, NANOG, and SOX2 via its demethylase activity targeting H3K27me2/3 [77]. Moreover, chemotherapy activates the A2BR/MAPK signaling pathway, which facilitates the nuclear recruitment of the chromatin remodeling factor SMARCD. In concert with p300, KDM6A further enhances the expression of these pluripotency factors, thereby contributing to the maintenance of cancer stemness [73].

KDM6B has 88% sequence homology with KDM6A [213]. KDM6B drives downstream gene transcription via H3K27me2/3 demethylation, promoting the expression of genes such as *snail*, which induces epithelial-mesenchymal transition (EMT) [82]. KDM6B upregulates inflammatory mediators, including IL-6, IL-1β and TNF-α to enhance antitumor immunity by promoting the polarization of macrophages toward the M1 phenotype in breast cancer [84]. Although KDM6A and KDM6B target the same histone modification sites, they exhibit distinct functional profiles in the regulation of histone methylation. KDM6A prefers to interact with the MLL, an H3K4 methyltransferase, while KDM6B antagonizes the H3K27 methyltransferase activity of EZH2 [79, 80]. The differential associated partners highlight the individual function of KDM6A or KDM6B on epigenetic regulation.

In luminal breast cancer, E2 induces EZH2 phosphorylation in endocrine sensitive cells, leading to its removal from chromatin and preventing further H3K27 methylation. Thereafter, KDM6B co-localizes with ER at enhancers and exerts its H3K27 demethylase activity to activate BCL2 expression [80]. However, in anti-endocrine-resistant cells, the significantly enhanced HER2 signaling drives EZH2 phosphorylation in an estrogen independent manner, leading to a global decrease in H3K27me3 levels and constitutive activation of ER due to the overexpression of BCL2, that circumvents the requirement of KDM6B at the enhancer region [80]. Overall, these findings demonstrate that KDM6B mediated demethylation and transcriptional activation are mechanistically based on methylation by EZH2 at the same genomic locus. KDM6B facilitates the expression of downstream genes by counteracting EZH2-mediated transcriptional repression. The research revealed that dependency on the KDM6B-IGFBP5 axis enhances the apoptotic response to PI3K/AKT inhibitor treatment [79]. KDM6B facilitates the expression of downstream IGFBP5, through counteracting EZH2-mediated transcriptional repression via its demethylase activity. Inhibition of IGFBP5 has been demonstrated to activate the IGF1R pathway, thereby promoting the PI3K-AKT signaling cascade [49, 192]. Additionally, PI3K phosphorylates EZH2, which enhances the synergistic action with KDM6B to modulate gene expression [79]. The imbalance between KDM6B and EZH2 can alter the methylation status of H3K27, thereby affecting transcriptional signatures and ultimately leading to adverse outcomes. An important key to understanding this phenomenon lies in elucidating the fundamental epigenetic mechanisms and the signature of epigenetic marks on chromatin.

#### KDM7

The KDM7 family, comprising members KDM7A (JHDM1D/KIAA1718), KDM7B (PHF8), and KDM7C (PHF2). They usually act as active histone demethylases, carrying a conserved PHD domain and the JmjC domain. In comparison, KDM7C features a histidine-to-tyrosine substitution within the third iron-binding residue of the JmjC domain (Fig. 1) [157]. Several studies proposed the demethylase activities of KDM7A and KDM7B target multiple histone residues, including H3K9me1/2, H3K27me2, and H4K20me1 [222–224]. KDM7C was initially identified for its demethylase activity on H3K9me1 [225]. Unexpectedly, it has been shown that histidine-to-tyrosine mutation in KDM7C affects its demethylation activity on H3K9me2, which relies on phosphorylation by protein kinase A (PKA) [226, 227]. Additionally, KDM7C has been shown to demethylate H4K20me3, a capability not shared by KDM7A or KDM7B [228].

KDM7B has been the most extensively studied member of the KDM7 family in various cancers, particularly with respect to its roles in oncogenesis [229, 230]. Structurally, the flexible linker between the PHD and JmjC domains of KDM7B is crucial for its enzyme activity. The flexible linker enables the PHD domain to preferentially bind to H3K4me3, thereby facilitating demethylation of repressive histone marks by the JmjC domain [223]. In histone methylation regulation, KDM7B not only erases repressive methyl marks but also maintains high levels of H3K4me3, a histone modification associated with transcriptional activation [90, 231]. In breast cancer, KDM7B exerts its oncogenic function through its demethylase activity targeting H3K9me1/2, H3K27me2, and H4K20me1, thereby upregulating CCNA2 expression to facilitate cell cycle progression [89]. Additionally, KDM7B promotes the expression of SNAI1 and ZEB1 by both its demethylase activity and maintenance of high H3K4me3 levels, contributing to an EMT-like process [90].

KDM7A remains relatively understudied. However, an increasing number of studies have also provided evidence that KDM7A can act as an oncogene in breast cancer. In TNBC, KDM7A promotes the expression of MKRN1 through its demethylase activity. Consistent with this, compound 4, which inhibits the binding of KDM7A to H3K27me3, reduces MKRN1 expression, leading to decreased cellular stemness and increased expression of cell cycle arrest markers, such as p16, p21, and p27 [87]. In addition, KDM7A enhances tumor invasion and metastasis by increasing RHOJ expression through the removal of H3K9me2 and H3K27me2 [85].

Compared with KDM7A and KDM7B, KDM7C exhibits a unique demethylation activation pattern and targeted histone residues [226, 228]. The dynamics of KDM7C expression in the context of tumor remain poorly understood. It is located at 9q22, a region that is frequently deleted in several types of cancer including breast cancer, which supports its potential role as a tumor suppressor [232]. Upon phosphorylation by PKA, KDM7C activates to demethylate H3K9me2/3, thereby maintaining the epithelial state and reversing the EMT process, ultimately suppressing breast cancer progression [227].

### KDM family proteins participate in non-histone demethylation

Histone demethylation by KDM family has been extensively studied, revealing their essential roles in the regulation of gene expression and chromatin dynamics. However, the demethylation activities of KDMs on non-histone substrates has emerged as a critical regulatory mechanism in physiological and pathological processes [233]. In breast cancer, increasing evidence has demonstrated the significant impact of non-histone methylation on disease progression, highlighting the importance to understand the precise post-translational regulations of different KDM family members in this context (Fig. 3).

#### KDM-induced demethylation influences protein stability

SET and MYND Domain-Containing Protein 2 (SMYD2) is a lysine methyltransferase that has been shown to directly methylate EZH2 at lysine 307 (K307) in breast cancer. This methylation inhibits the ubiquitination of EZH2, thereby protecting it from proteasome-mediated degradation. Conversely, KDM1, a demethylase, can counteract the methylation of EZH2 K307 mediated by SMYD2. By promoting the degradation of EZH2, KDM1 alleviates EZH2-induced transcriptional repression of tumor suppressor genes, including *RASSF1*, *SIAH1*, and *AXIN2* [19]. Taken together, KDM1 suppresses breast cancer tumorigenesis and metastasis by attenuating EZH2 stability through demethylation.

HIF1α stability is regulated by various post-translational modifications, including methylation, hydroxylation, and acetylation. Von Hippel-Lindau protein (VHL), a component of the E3 ubiquitin ligase complex, recognizes hydroxylated and acetylated HIF1α and promotes its ubiquitination and proteasomal degradation [17]. KDM1 demethylates HIF1α at lysine 391. During the demethylation, KDM1 generates H₂O₂ to inhibit the hydroxylase activity of PHD2, thereby suppressing HIF1α hydroxylation. Additionally, KDM1 cooperates with the metastasis associated 1 (MTA1) and NuRD complex to deacetylate HIF1α. By modulating HIF1α hydroxylation and acetylation, KDM1 abolishes VHL-mediated ubiquitination of HIF1α to stabilize its protein levels, ultimately enhancing HIF1α-driven angiogenesis in breast cancer [17]. Moreover, KDM6B accelerates the ubiquitin-mediated degradation of β-catenin via its demethylase activity. Specifically, KDM6B promotes the intranuclear degradation of β-catenin, thereby preventing the polarization of macrophages towards the M2 phenotype in breast cancer [83].

#### KDM-induced demethylation modulates protein activity

Different methylation sites on histones are well-known to induce either transcriptional activation or repression. Similarly, methylation at distinct sites on the p53 protein can exert distinct effects on its activity. Methylation at p53 K370 by Smyd2 reduces its DNA-binding capacity to inhibit p53-mediated transactivation [234]. In contrast, methylation at p53 K372 by Set9 significantly enhances p53 protein stability to promote its function [235]. Different KDM family members selectively target distinct methylation sites on p53. KDM1 targets K370 to demethylate p53, thereby promoting its activity [236]. In breast cancer, KDM3A targets p53 K372 methylation, suppressing the tumor-suppressive function of p53, leading to the downregulation of pro-apoptotic proteins PUMA and NOXA [39].

Phosphorylation of estrogen receptor (ER) is a critical post-translational modification that enhances its transcriptional activity [237]. Recent research indicates that KDM2A, an epigenetic regulator, is upregulated in response to endocrine disruptors BPA and BPS. The increased expression of KDM2A may facilitate the phosphorylation of ERα by demethylating specific lysine residues, thereby enhancing ERα phosphorylation. This process activates the ER signaling pathway, thus promoting tumorigenesis in luminal breast cancer [25].

DNA topoisomerase 2-binding protein 1 (TOPBP1), an essential allosteric activator of ATR, facilitates ATR activation through its interaction with the RAD9-HUS1-RAD1 (9–1-1) complex. KDM7B demethylates TOPBP1 at lysine 118 promotes the binding of TOPBP1 to RAD9 [238]. Importantly, disruption of the KDM7B-TOPBP1 axis inhibits ATR activity to increase chromosomal instability, thus suppressing tumorigenesis and enhancing a tumor-specific susceptibility to PARP inhibitors (PARPi) in breast cancer [239].

### KDM family proteins function in a demethylase-independent manner

#### KDM2

Basally active estrogen-repressed (BAER) enhancers are highly active under basal conditions but are transcriptionally repressed by estrogen (E2) through ERα. Specifically, ERα is recruited to BAER enhancers in a manner dependent on its DNA-binding domain (DBD), which interacts with KDM2A. Subsequently, KDM2A recruits the E3 ubiquitin ligase NEDD4 to these enhancers, leading to ubiquitination and degradation of RNA polymerase II (Pol II). This process effectively inhibits the transcription of downstream target genes, thereby mediating the repressive effects of E2 on BAER enhancer activity [24].

KDM2B (FBXL10) interacts with ERRα, and stabilizes ERRα protein levels by reducing its poly-ubiquitylation and promoting its mono-ubiquitylation. KDM2B also enhances ERRα enrichment at the promoter region of its target genes to increase ERRα-mediated transactivation, thereby promoting the cell proliferation in breast cancer [30].

#### KDM5

Composed of multiple domains, KDM5 harbors functional capabilities in its AT-rich interaction domain (ARID) and plant homeodomain (PHD) that operate independently of the demethylase activity associated with its JmjC domain [114]. In transcriptional regulation, PHD can activate transcription independently of histone demethylase activity [240]. During the progression of TNBC, the selective binding of H3K4me0 by the PHD1 domain of KDM5B, in conjunction with its histone demethylase activity, is crucial for suppressing the invasion and metastasis [188]. Additionally, KDM5A regulates the expression of genes like tenascin-C (TNC) independently of its demethylase activity. Instead, the N-terminal ARID, which binds to DNA, and the C-terminal LXCXE motif, which interacts with Rb and p107, are critical for modulating TNC expression and promoting cell invasion and metastasis. Consistent with this, mutating the demethylase activity domain (H483A) does not alter TNC expression levels, highlighting the non-catalytic functions of KDM5A in gene regulation [241]. These studies indicate that KDM5 regulates gene expression through multiple mechanisms, which may play important roles in diverse biological processes.

KDM5 typically acts as a transcriptional repressor by removing transcriptional activation signals through H3K4 demethylation [184]. However, in specific transcription start sites (TSS), the demethylase activity of KDM5 is constrained. Instead, KDM5 recruits other proteins to form a transcriptional activation complex, thereby promoting downstream transcription. KDM5A is a component of the transcriptional activation complex EMSY and is recruited to the TSS by ZNF131. Within this complex, KDM5A recognizes H3K4me3 and collaborates with Sin3/HDAC to maintain histone-mediated transcriptional activation signals. Although histone methylation is dynamically regulated and KDM5A functions as a demethylase during subsequent demethylation events, its non-enzymatic role in the transcriptional activation complex is also significant at specific temporal phases [50]. KDM5A cooperates with CBP/p300 to enhance ERα-dependent transactivation independent on its demethylase activity (Fig. 7), KDM5A also increases the stability of the ErbB family receptors EGFR and HER2 and subsequent PI3K-AKT activation regardless of its demethylase activity. These two pathways regulation indicates that KDM5A is involved in tamoxifen-resistance [49]. In luminal breast cancer, the JmjC domain of KDM5C directly interacts with the AF2 domain of ERα, which inhibits the demethylase activity of KDM5C, thereby abrogating its H3K4 demethylation and the resultant transcriptional repression. Meanwhile, at active enhancers bound by ERα, KDM5C collaborates with ZMYND8 to recruit the P-TEFb complex, functioning as a co-activator to promote the transcriptional activation of estrogen/ERα target genes [66].

In eukaryotes, the length of the 3′UTR influences the ability of mRNA to interact with microRNAs, thereby modulating mRNA stability and translation efficiency [242]. In the MCF-7 cell line, treatment with a KDM5 inhibitor resulted in increased global levels of H3K4 methylation but did not affect the length of the 3′UTR of downstream genes such as CCND1 [243]. Overall, KDM5 functions in an enzyme-independent manner to regulate the length of certain gene mRNAs, contributing to the regulation of breast cancer.

#### KDM6

The highly conserved TPR domain of KDM6A, which mediates critical protein–protein interactions, is essential for its demethylase-independent transcriptional regulation. Through this TPR domain, KDM6A also named as ubiquitously transcribed tetratricopeptide repeat on chromosome X (UTX) forms complexes with a variety of histone-modifying proteins, including other members of KDM family [78], the MLL family [221], the KAT family [244], as well as non-histone ubiquitin ligases. In these complexes, KDM6A functions as a scaffold protein rather than as a histone demethylase, orchestrating coordinated regulation of gene expression. KDM6A recognizes MLL4 and recruits KDM1A, inducing H3K4me2 demethylation and leading to competition between MLL4 and KDM1A (Fig. 7). This epigenetically silences EMT-related transcription factors, inhibiting breast cancer stem cell characteristics [78]. It has been reported that an epigenetic crosstalk occurs at enhancers between the KDM6A-MLL4 complex and the histone acetyltransferase p300. UTX facilitate recruitment and cooperativity of MLL4 and p300, driving a feedforward regulatory loop in setting up active enhancer landscapes. While KDM6A demethylase activity is not required for setting up active enhancers [245]. UTX regulates mesoderm differentiation and Brachyury expression independent of its enzymatic activity [246].

KDM6B interacts with PHF20 and concurrently recruits the E3 ubiquitin ligase Trim26 through its N-terminal domain. This interaction leads to the degradation of PHF20 via K48-linked polyubiquitination. As a result, KDM6B inhibits PHF20's activation of Oct4, thereby preventing somatic cell reprogramming from a differentiated to a pluripotent state [247].

### Upstream regulation of KDM family proteins

Have established that KDMs participate in modulating a series of transcription factors-induced gene transcription through demethylase dependent or independent manner to exert pivotal roles in regulation of cancer progression. KDMs are abnormally expressed in cancers and commonly considered as the potential therapeutic targets for cancer treatment. Thus, clarification of the upstream regulation of KDMs would be important for providing new strategies for designing the effective drugs.

#### Post-translational modifications of KDMs

KDM family enzymes exert their functions by modulating the methylation of proteins. Meanwhile, KDMs themselves also undergo diverse post-translational modifications (PTMs), including ubiquitination, phosphorylation, and lactylation [94, 248, 249], which emerge as critical contributors and therapeutic targets in cancers [250–252]. Such PTMs at specific sites on KDMs in breast cancer cells enhance their functional diversity and contribute to breast cancer progression (Table 2).

Ubiquitination at distinct sites regulates the stability of KDMs in breast cancer cells. HDAC5 physically interacts with KDM1A (LSD1) and recruits USP28 to reduce KDM1A polyubiquitination, to prevent its proteasomal degradation [15]. KDM1A undergoes K63-linked polyubiquitination, and research has shown that OTUD7B deubiquitinates KDM1A at K266/277, thereby stabilizing its expression and maintaining its dynamic regulation during the breast cancer cell cycle [16]. Fbxo22 exerts tumor-suppressive effects in breast cancer by degrading KDMs [51, 91]. In ER-positive breast cancer, Fbxo22-mediated degradation of KDM4B maintains the antagonistic activity of ER [91]. In TNBC, Fbxo22 promotes the degradation of KDM5A, thereby upregulating the expression of the downstream tumor suppressor gene p16 and inhibiting tumor invasion [51]. TRIM11 is a ubiquitin E3 ligase that targets and promotes the degradation of KDM5C, thus enhancing the expression of the downstream MCAM adhesion molecule and facilitating the migration of breast cancer cells [68].

Multiple kinase signaling systems orchestrate breast cancer progression by modulating the phosphorylation of KDMs [253], a mechanism that drives oncogenic pathways in breast cancer. In multiple breast cancer cell lines, activation of the PI3K/AKT signaling pathway induces cytoplasmic relocalization of KDM5A via phosphorylation. This spatial redistribution disrupts its chromatin association, driving genome-wide transcriptional activation [92].

KDM3A is phosphorylated and activated by multiple kinases to promoter breast cancer development [38, 41]. ACK1-mediated phosphorylation of KDM3A at the Tyr-1114 site, which significantly activates its H3K9me2 demethylase activity, further promotes the transcriptional activation of the downstream oncogene HOXA1 (Table 2) [38]. Additionally, KDM3A is tyrosine-phosphorylated by JAK2 to act as a coactivator for STAT3, thereby exerting increased cancer cell growth and motility [41]. Phosphorylation is a critical regulator of KDM activity, while it also serves as a prerequisite for ubiquitination, integrating crosstalk among PTMs [93]. CDK9-mediated phosphorylation of KDM1A is a precondition for subsequent RNF20-mediated K29-linked ubiquitination (Table 2). In breast cancer, the CDK9- and RNF20-dependent LSD1 stabilization is essential for H3K4 demethylation. This process ultimately contributes to transcriptional repression and immunosuppression in breast cancer, thereby promoting tumor progression [93].

Lactylation modification, an emerging frontier in cancer research, has also been found to participate in PTM of KDMs. Studies on melanoma have revealed that in a lactic acid accumulation microenvironment, the K503 site of KDM1 undergoes lactylation modification, which not only inhibits TRIM21-mediated ubiquitination but also enhances the interaction between KDM1A and FosL1 (Table 2). Together, these two proteins inhibit TFRC-mediated ferroptosis, thereby promoting the survival of drug resistant melanoma cells [94]. Collectively, these findings suggest that the lactylation of KDM1A may represent an important potential factor in breast cancer treatment resistance.

#### Epigenetic regulation of KDM expression

The epigenetic factors or drugs involved in RNA or DNA modification are critical for regulation of KDM expression and are closely associated with tumorigenesis in breast cancer [95, 96]. YTH N6-methyladenosine RNA-binding protein 2 (YTHDF2) recognizes N6-methyladenosine (m^6^A) methylation in KDM1 mRNA to facilitate its stability to enhance KDM1 expression, thereby promoting breast cancer progression [95]. Ginsenoside (Rg3) modulates the expression of KDM5A by altering the CpG island methylation status of KDM5A DNA, subsequently suppressing cell proliferation and invasion in breast cancer (Table 2) [96].

Non-coding RNA has been shown to regulate KDM expression and activity. MicroRNAs (miRNAs) are endogenous small non-coding RNAs that regulate gene expression by binding to the 3′ UTR of the mRNA targets [254]. Increasing evidence shows a deregulation of miRNA expression in human cancer, indicating that they are potential biomarkers for cancer diagnosis and prognosis, as well as therapeutic targets or tools [255]. miR-486 as one of the mostly downregulated miRNAs in breast cancer regulates JARID1B/KDM5B expression in breast cancer cells. miR-486 induced JARID1B downregulation is accompanied by accumulation of DNA damage and enhanced radiosensitivity [97]. In addition, miR-22 has been shown to suppress PHF8/KDM7B expression (Table 2). Within the MYC/miR-22/PHF8 axis, MYC post-transcriptionally up-regulates PHF8 by repressing miR-22, thereby positioning PHF8 as a downstream effector of MYC. Consequently, PHF8 facilitates MYC-driven proliferation and migration of breast cancer cells [90]. In *Drosophila*, long hairpin RNAs was identified as Lsd1-interacting non-coding RNAs (LINRs) from fly ovaries by hLsd1 specific RNA immunoprecipitation sequencing (RIP-seq) experiments to regulate stem cell/progenitor differentiation, although its function in cancers is unknown [256].

Long intergenic non-coding RNAs (lincRNAs) regulate chromatin states and epigenetic inheritance. The studies have demonstrated that lincRNA HOTAIR acts as a scaffold protein for at least two distinct epigenetic complexes. A 5′domain of HOTAIR binds polycomb repressive complex 2 (PRC2), whereas a 3′domain of HOTAIR binds the KDM1A/CoREST/REST complex. The ability to tether two distinct complexes enables RNA-mediated assembly of PRC2 and KDM1A complexes and coordinates targeting of PRC2 and KDM1A to chromatin for coupled histone H3 lysine27 methylation and lysine4 demethylation (Table 2) [98]. In gastric cancer cell, Linc01446 interacts with KDM1A and recruits KDM1A to the promoter of *Ras-related dexamethasone-induced 1* (*RASD1*) gene, thereby suppressing *RASD1* transcription (Table 2) [99].

**Table 2:** Upstream regulation of KDM expression

	Types	Upstream regulator	Target KDMs	Regulatory mechanisms	Ref
PTMs	ubiquitination/deubiquitination	HDAC5-USP28	KDM1A	HDAC5-USP28 removes polyubiquitin from KDM1 to block proteasomal degradation	[15]
		OTUD7B	KDM1A	OTUD7B stabilizes KDM1 by cleaving K63-linked polyubiquitin at K266/277	[16]
		Fbox22	KDM4B	Fbxo22 mediates the degradation of KDM4B to sustain SERM activity	[91]
			KDM5A	Fbxo22 promotes the degradation of KDM5A to up-regulate the tumor suppressor p16	[51]
		TRIM11	KDM5C	TRIM11 promotes the degradation of KDM5C to enhance the expression of MCAM	[68]
	phosphorylation	PI3K/AKT signaling pathway	KDM5A	PI3K/AKT-mediated phosphorylation triggers cytoplasmic relocalization of KDM5A to drive genome-wide transcriptional activation	[92]
		ACK1	KDM3A	ACK1 phosphorylates KDM3A at Tyr1114 to activate H3K9me2 demethylation and transactivate HOXA1	[38]
		CDK9, RNF20 (ubiquitination)	KDM1A	CDK9 phosphorylation primes KDM1A for RNF20-catalyzed K29 ubiquitination that silences H3K4me and drives immunosuppression	[93]
	lactylation	lactic acid accumulation microenvironment	KDM1A	KDM1A K503 lactylation blocks TRIM21 ubiquitination and enhances KDM1A FosL1 binding	[94]
Epigenetic regulation	RNA modification	YTHDF2	KDM1A	YTHDF2 recognizes m6A-marked KDM1 mRNA to stabilize and upregulate KDM1 expression	[95]
	DNA modification	Ginsenoside (Rg3)	KDM5A	Rg3 remodels CpG methylation of the KDM5A promoter to modulate its expression	[96]
	miRNAs	miR-486	KDM5B	miR486 silences JARID1B to accumulate DNA damage and enhance radiosensitivity	[97]
		MYC-miR-22	KDM7B	MYC represses miR-22 to up-regulate PHF8 and promote breast cancer cell proliferation and migration	[90]
	lincRNAs	HOTAIR	KDM1A	HOTAIR scaffolds PRC2 and LSD1 to chromatin to couple H3K27me and H3K4me removal	[98]
		Linc01446	KDM1A	Linc01446 interacts with LSD1 and delivers it to the RASD1 promoter to silence transcription	[99]

Taken together, the above studies suggest that the upstream regulation of KDMs mainly through post-transcription modification, DNA/RNA modification, and non-coding RNA regulation. It would help us deeply understand the reason for abnormal expression levels of KDMs in cancers, thereby providing more effective cancer therapy strategies.

## KDM family in breast cancer hallmarks and biological functions

Individual KDM family members execute highly context-specific programs that orchestrate virtually every hallmark of malignancy. KDMs mainly exert their essential biological functions in DNA damage response, cell-cycle transit regulation, cell senescence, therapeutic resistance, tumor metabolism, tumor stem-like plasticity, and reprogramming of tumor microenvironment (Fig. 6).

### DNA damage response

Genomic instability is a hallmark of cancer and underpins traditional cancer therapies such as radiotherapy and DNA-damaging chemotherapy [257]. Advanced therapeutic approaches, like PARP inhibitors used in breast cancers with BRCA1 or BRCA2 mutations, target tumor specific DDR, thereby minimizing collateral DNA damage to normal tissues [258]. Emerging evidence indicates that KDMs influence breast cancer progression and radiotherapy sensitivity through the regulation of DDR pathways.

KDM4 is intimately linked to the DNA damage response (DDR) in breast cancer [259–261]. Following DNA damage, KDM4A is degraded via ubiquitination mediated by the E3 ligases RNF8 and RNF168. This degradation exposes H4K20me2 sites, enhancing p53 accessibility to DNA damage sites and promoting p53-mediated repair (Fig. 6) [259]. Additionally, in breast cancer, KDM4B overexpression promotes LINE-1 activation by demethylating H3K9me3, leading to sustained LINE-1 retrotransposition. This process induces DNA double-strand breaks (DSBs), thereby exacerbating genomic instability (Fig. 6). Moreover, the loss of H3K9me3 modification itself has been shown to impair DSB repair and increase radio sensitivity, underscoring the role of H3K9 methylation in maintaining genomic integrity [260]. In contrast, it is suggest that inhibiting KDM4B phosphorylation with RSK inhibitors abrogates its accumulation at DSB sites, suppressing repair and enhancing ionizing radiation sensitivity in breast cancer cells [261]. Transcriptomic analyses reveal that high KDM5B expression in breast cancer patients often correlates with low levels of miR-381 and miR-486. KDM5B, enriched at damage sites through PARP1 and macroH2A1.1 dependent mechanisms, facilitates non-homologous end joining (NHEJ) and homologous recombination (HR) by recruiting repair proteins like Ku70/80 and BRCA1. In MCF7 cells, miR-486 overexpression reduces KDM5B to increase DNA damage accumulation and sensitize breast cancer cells to radiotherapy [97].

In summary, KDMs regulate DNA damage and repair, highlighting the therapeutic potential of targeting KDM family members to enhance the efficacy of radiotherapy in breast cancer.

### Cell cycle

KDMs regulate breast cancer progression by modulating cell-cycle-related proteins to influence the cell cycle process. Research shows KDM2B impacts cell fate in multiple processes across different cell lines [33]. In MCF7, T47D, and MDAMB—231, KDM2B knockdown arrests the G1 phase, indicating it contributes to transition from G1 to S in all. However, its ability to induce senescence and apoptosis varies. In T47D cells KDM2B knockdown mainly triggers senescence, whereas in MCF7 and MDA-MB-231 cells, it predominantly induces apoptosis [33]. This discovery highlights regulation role of KDM2B in cell fates and offers insights into its functional differences across cell lines.

As a downstream gene of both HIF-1α and ER, KDM4B modulates the expression of cell cycle genes like *CCND1*, *CCNA1*, and *WEE1* via epigenetic regulation in hypoxic conditions [45]. Moreover, KDM4B forms a feed-forward loop to modulate the E2 response. In MCF-7 cells, KDM4B promotes estrogen-induced cell proliferation by facilitating the G1 to S progression [46].

Cyclins are a class of proteins that bind to cyclin-dependent kinases (CDKs) at different cell cycle stages to drive the cell cycle. KDM5 modulates their expression across diverse transcriptional regulation planes [197, 243]. In modulation of 3'UTR length, KDM5B is associated with shortened CCND1 mRNA that lacks miRNA binding sites, resulting in mRNA stabilization and translational activation of CCND1 (Fig. 6) [243]. In regulation of miRNAs including let-7e, which targets to CCND1, KDM5B binds to the let-7e promoter to inhibit its expression and enhance CCND1 expression. Consequently, overexpression of cell cycle promoter contributing to breast cancer progression [197].

Cyclin-dependent kinase inhibitors, such as the p16 (CDKN2A) and p21 (CDKN1A) genes, play crucial roles in suppressing cell cycle progression [262]. Recent studies have highlighted the expression levels of p16 and p21 can serve as biomarkers to predict the efficacy of CDK inhibitors [263]. Immunohistochemical analysis of tumor samples from 176 patients with invasive ductal carcinoma revealed that higher tumor grades were associated with increased expression of KDM5B, whereas a negative correlation was detected between P16 and KDM5B expression [264]. ER downstream genes TFAP2C and Myc are overexpressed in breast cancers associated with poor prognosis [265, 266]. Dysregulation of these genes results in *p21* loss in MCF-7 cells, while the incorporation of KDM5B significantly enhances this effect by removing H3K4me3 marks on *p21* promoter [56]. Thus, the TFAP2C-Myc-KDM5B complex drives cell cycle progression by directly repressing CDKN1A, contributing to tumorigenesis and endocrine therapy failure. KDM7B also involved in cell cycle regulation. It positively correlated with expression of CCNA2 and the histological grade of breast cancer (Fig. 6) [89].

### Cell senescence and death

Senescence is a tumor-suppressive mechanism marked by irreversible cell cycle arrest [267, 268]. KDMs are demonstrated to promote breast cancer growth by inhibiting tumor cell senescence [20]. *TBX2* is an anti-senescence gene, promoting cell growth and survival through repression of Tumor Suppressor Genes (TSGs), such as *NDRG1* and *CST6*. Research identifies KDM1 as novel interactors of TBX2 to suppress expression of *NDRG1* and *LINC00111*. Altogether, inhibition of KDM1 leads to a promising treatment for TBX2-addicted breast tumors [20].

P53 is a critical mediator of cellular senescence [269] and it is reported that multiple KDM members are involved in p53-induced senescence [270, 271]. In fibroblasts, overexpression of KDM2A mediates p53-dependent senescence, facilitating the release of cytokines. These cytokines stimulate KDM2A expression and transform normal fibroblasts into cancer-associated fibroblasts (CAFs), which ultimately contribute to breast tumor growth [270]. KDM4A plays a crucial role in cellular senescence by forming a complex with Fbxo22, a senescence-associated E3 ligase. This complex specifically targets methylated p53 for degradation. During the late phase of senescence, the Fbxo22-KDM4A complex is essential for regulating the induction of p16 and the senescence-associated secretory phenotypes (SASP), thus participating in the overall regulation of cellular senescence (Fig. 6) [271]. Metformin was predicted to regulate the process of aging as it targets and suppresses the demethylation activity of KDM6A to elevate global levels of H3K27me3 in non-diabetic breast cancer patients [272].

KDMs suppress tumor cell apoptosis through diverse mechanisms to facilitate tumor development [40, 80, 88, 273]. Caloric restriction (CR) initially exerts inhibitory effects on leukemia. However, leads to disease relapse over time. This study found that under CR conditions, combining KDM1 inhibition increases leukemia cells' apoptosis sensitivity. Furthermore, in TNBC patient-derived xenograft (PDX) models, CR combined with an KDM1 inhibitor further reduces tumor volume and sensitizes TNBC to TRAIL-induced apoptosis [273]. KDM3A induces apoptosis by demethylating H3K9me1/2 and promoting the transcriptional activation of the pro-apoptotic genes BNIP3 and BNIP3L (Fig. 6) [40]. BCL2 is a notable anti-apoptotic protein that inhibits apoptosis [274]. Different KDM members involve in regulatory of BCL2 expression [80, 88]. In tamoxifen-sensitive cells, BCL2 activation involves recruitment of KDM6B to the promoter and detachment of EZH2. While, in endocrine therapy-resistant cells, BCL2 is constitutively active independently of KDM6B [80]. BAD is a BCL2 family pro-apoptotic protein whose phosphorylation inhibits its function. Research shows that KDM7A knockdown promotes breast cancer cell apoptosis by reducing BCL2 expression and BAD phosphorylation [88].

### Cell stemness

Cancer stem cells (CSCs) are a small group of cells with self-renewal, unlimited proliferation, and multipotent differentiation. They strongly resist conventional chemotherapy, and are considered the root cause of tumor occurrence, development, metastasis, and recurrence [275, 276]. Recently, increasing research has focused on the role of stemness in breast cancer. Breast cancer stem cells (BCSCs) not only originate from mutations or overactivation of the self-renewal ability of normal stem cells but also a consequence of the de-differentiation of cancer cells caused by somatic mutations or microenvironmental factors during treatment [277]. KDM family enzymes regulate breast cancer stemness (BCS) through epigenetic regulation, modulating the activity of signaling pathways that sustain stemness and the expression of stemness markers and genes.

KDM1 is overexpressed in various CSCs, including breast cancer. It modulates key signaling pathways (WNT/β-catenin, Notch, Sirt1, TGF-β1, JAK/STAT3) and core stemness-related transcription factors (SOX2, OCT4, Sall4, c-MYC) through diverse mechanisms. These actions suggest that KDM1 plays a crucial role in maintaining BCSCs [278]. In TNBC upregulation of KDM1 is linked to poor prognosis. KDM1 inhibits expression of METTL14, which is essential for recognizing structure-specific N6-methyladenosine (m6A) motifs and guiding METTL3 in m6A deposition [279]. Notably, research reveals that low METTL14 expression also predicts unfavorable outcomes [280]. By suppressing METTL14, KDM1 reduces m6A modification levels to stabilize YAP1 mRNA and increase its expression, thereby driving cancer stem cell self-renewal by activating the YAP1 pathway [130]. The retinoblastoma binding protein (RBBP) family is noted for its distinctive epigenetic roles. Family members, RBBP4 and RBBP7 form several epigenetic complexes including PRC2, NuRD, and NURF, to mediate histone modification and chromatin remodeling [281]. In breast cancer, RBBP7 enhances stemness by recruiting the NuRD complex subunit KDM1 to remove repressive H3K9me3 marks from promoters of stemness-related genes (*SOX9*, *SOX2*, *OCT4*, *CCND1*) and boost their expression [104].

In breast cancer cell lines, KDM2B knockdown reduces the expression of the stem cell markers ALDH and CD44 while increasing CD24 levels, indicating its key role in cancer stem cell (CSC) maintenance. Mechanistically, KDM2B cooperates with EZH2 to transcriptionally suppress miRNAs like miR-101, miR-181, miR-200b, and miR-203, which are normally downregulated in both normal and BCSCs. Moreover, these miRNAs target PRC1 and PRC2 subunits, including BMI1, RING1B, SUZ12, and EZH2 itself. Thus, KDM2B inhibition promotes these miRNAs to block PRC1 and PRC2 activity and ultimately restricting BCSC self-renewal [33].

OCT4 is crucial for BCSC stemness maintenance and shares a mutual regulatory relationship with KDM family members. As a transcription factor, OCT4 upregulates KDM3A and KDM4C [282], meanwhile KDM6A is essential for maintaining OCT4-induced transcription [77]. In ESCs, KDM3A and KDM4C drive stemness-related gene expression through H3K9 demethylation. KDM3A removes H3K9Me2 from the promoter regions of Tcl1, Tcfcp2l1, and Zfp57, while KDM4C positively regulates Nanog through its H3K9Me3 demethylation. ESCs differentiate and lose self-renewal and pluripotency after depletion of KDM3A or KDM4C (Fig. 6) [282]. KDM6A acts as an Oct4 co-activator, and stabilizing this interaction in the tumor hypoxic microenvironment. It mediates H3K27me3 demethylation at promoters of OCT4 target genes such as *KLF4*, *NANOG*, and *SOX2* to preserve BCSC stemness (Fig. 6) [77].

Given the crucial role of KDM4 in cell stemness, researchers have developed the KDM4 inhibitor QC6352. By inhibiting KDM4, QC6352 reduces the expression of EGFR in cancer stem cells (CSCs), thus suppressing tumor growth and metastasis. In preclinical studies, QC6352 has shown significant therapeutic effects on TNBC and offers a novel treatment strategy [108]. However, conflicting views exist on KDM4's role in stemness, a recent study has demonstrated that KDM4B diminishes the expression of stemness-associated transcription factors Nanog and SOX2 [283]. Overall, these findings highlight the complicated roles of KDM4 in breast cancer stemness, suggesting that further research is needed to fully understand their therapeutic potential.

The studies have also shown that KDM3A up-regulates the transcription of pluripotent factors Sox2 and Nanog and Bcl-2 to promote ovarian cancer progression [284], KDM4B induced by ETBF epigenetically activated Nanog expression through H3K9me3 demethylation on its promoter, to mediate cell stemness in colorectal cancer [285]. KDM4C forms a positive feedback loop with ALDH1A3 which is a stemness biomarkers in gastric cancer [286]. While the functions of these KDM members in breast cancer stemness (BCS) maintenance is still elusive, it indicates that they may play crucial roles in BCS.

### Tumor metabolic reprogramming

Metabolic reprogramming is a hallmark of cancer, providing tumors with essential bioenergetic substrates and enabling tumor cells to adapt to the malignant microenvironment [287, 288]. Metabolic alterations in breast cancer, including glucose, lipid, and amino acid metabolism, are closely linked to cancer progression and drug resistance [289]. Beyond regulating the expression of key factors in multiple metabolic pathways, KDMs are also sensitive to metabolic changes, as various metabolites directly impact their enzyme activities [290]. This interplay underscores the critical role of KDMs in breast cancer metabolic regulation.

#### Lipid metabolism

Lipid serves as a vital nutrient in cellular metabolism. As a key component of metabolic reprogramming in cancer, lipid metabolic reprogramming enables tumor cells to generate energy through lipid oxidation, supporting their survival in adverse environments [291, 292]. Recent studies have identified FLAD1, a metabolic gene identified to be amplified in TNBC. As a key regulator of lipid metabolism, FLAD1 enhances the enzymatic activity of KDM1A toward H3K9me2, leading to increased expression of SREBP1, which is a critical transcription factor that governs lipid synthesis, and its activation results in elevated expression of lipid metabolic enzymes, including FASN, ACC1, and SCD. Collectively, the FLAD1/LSD1/SREBP1 axis reprograms lipid metabolism, thereby promoting TNBC proliferation and migration (Fig. 6) [293]. In MCF-7 and MDA-MB-231 cells, KDM5B upregulates key lipid metabolism genes such as FASN and ACLY via inhibiting AMPK, thereby promoting lipid metabolism reprogramming [63]. Additionally, KDM6 is involved in lipid metabolism-mediated antitumor effects. PPARα and NPC1L1 are both key factors in lipid metabolism. Research shows that combining fenofibrate (a PPARα agonist) with ezetimibe (an NPC1L1 inhibitor) induces cytoplasmic vacuolation and apoptosis in breast cancer cells via reprogramming lipid metabolism. Mechanistically, this combination activates the IRE1α/XBP1s pathway, which depends on KDM6B interaction to trigger the unfolded protein response, thereby significantly inhibiting breast tumor growth [294].

#### Amino acid metabolism

Proteins are another important nutrient in addition to glucose and fats. Among all amino acids, glutamate is extensively studied due to its critical roles in vivo and is gaining attention as a therapeutic target in cancer treatment [295].

Glutamine metabolism, a key energy source for tumor cells, produces α-ketoglutarate (α-KG) when overactivated, which provides energy to support tumor cell survival [296]. KDMs regulate glutamine metabolic reprogramming, providing tumor cells with the necessary support to survive under conditions of glucose and amino acid deprivation [297, 298]. GDH1 is a metabolic enzyme that converts glutamate into α-KG, which is not only a vital intermediate of the tricarboxylic acid (TCA) cycle but also a cofactor that modulates the activity of KDMs [297]. Under amino acid deprivation conditions, GDH1 either degrades via ubiquitination or translocates from mitochondria to the cytoplasm. This leads to a significant reduction in intracellular αKG levels, which inhibits the demethylase activity of KDMs. Consequently, histone methylation levels (H3K9me3 and H3K27me3) increase, thereby suppressing ribosomal protein gene expression. This epigenetic mechanism allows tumor cells to adapt to amino acid deprivation by reducing protein synthesis [297]. Glucose deprivation is a hallmark of the tumor microenvironment. This condition elevates the levels of mitochondrial cytochrome c oxidase II (MT-CO2) and flavin adenine dinucleotide (FAD), thereby activating KDM1. Moreover, KDM1-induced H3K9 demethylation promotes JUN transcription, which in turn facilitates the expression of glutaminase (GLS) and enhances glutaminolysis [298]. In TNBC, KDM2B modulates multiple metabolic pathways in concert with MYC and ATF4. In the serine-glycine-one-carbon (SGOC) metabolic pathway, KDM2B influences expression of crucial enzymes, PHGDH and MTHFD2. Additionally, it regulates glutamate metabolism by targeting enzymes like GCLC, GCLM, and GSS, which are essential for glutathione (GSH) to maintain cellular antioxidant capacity [32]. The coordinated overexpression of KDM2B, MYC, and ATF4 defines a distinct metabolic subtype, MPS2, characterized by heightened activity of the SGOC and glutamate metabolic pathways and associated with poor clinical outcomes (Fig. 6) [32]. These findings highlight critical role of KDM2B in metabolic reprogramming and position KDM2B as a promising therapeutic target in TNBC.

KDM4C interacts with ATF4, which is a transcriptional master regulator of amino acid metabolism, and epigenetically activates the serine-glycine synthesis pathway via H3K9 demethylation, to promote cancer cell proliferation [299]. Besides, KDM4C promotes HIF-1 induced breast cancer progression HIF-1 interaction with hypoxia response elements, leading to the activation of BNIP3, LDHA, PDK1, and SLC2A1 transcription, which encode proteins essential for metabolic reprogramming [47]. EMSY binds to the JmjC domain of KDM5B, suppressing its demethylase activity. This leads to an increase in H3K4me2/3 levels and promotes the expression of genes involved in methionine metabolic, thereby reshaping methionine metabolism in TNBC [57].

#### Tumor microenvironment reprogramming

The tumor microenvironment (TME) is a complex ecosystem shaped by the crosstalk between cancer cells, immune cells, stromal cells, and the extracellular matrix, along with soluble mediators and signaling molecules [300]. Beyond its critical role in tumor progression, TME emerges as advancing targets for immunotherapies [301]. In breast cancer, particularly TNBC, the combination of immune checkpoint inhibitors with cytotoxic chemotherapy serves as a standard therapeutic approach [302, 303]. Mounting evidence indicates that epigenetic alterations mediated by KDMs exert a significant influence on the reprogramming of the TME.

KDM1A plays dual roles in both innate and adaptive tumor immunity [304]. Inhibition of KDM1A reverses the immunosuppressive effects within the tumor microenvironment and is also emerging as an attractive therapeutic strategy in breast cancer [106]. KDM1 has been implicated in T-cell exhaustion [105]. Recent study elucidates the specific mechanism by which KDM1A inhibition in TNBC enhances CD8 + T-cell tumor infiltration. Suppression of KDM1A upregulates TFPI2, a tumor suppressor gene, leading to downregulation of matrix metalloproteinases (MMPs) and subsequently increasing chemokine levels that attract cytotoxic T cells within the tumor microenvironment [305]. Thus, in poorly immunogenic TNBC, inhibiting KDM1A can enhance antitumor immunity by promoting CD8 + T cells infiltration. Furthermore, KDM1A expression in TNBC is inversely associated with several antitumor immune factors, including cytotoxic T cell-attracting chemokines (CCL5, CXCL9, CXCL10) and PD-L1. Subsequent research has shown that inhibiting KDM1A increases H3K4me2 levels at proximal promoter regions of these chemokines to restore their expression and enhance immunotherapy efficacy [107]. Besides, KDM1A suppresses endogenous retrovirus (ERV) transcription via H3K4 demethylation, reducing interferon responsiveness and leading to epigenetic immunosuppression in breast cancer. Research reveals that RNF20-mediated ubiquitination and CDK9-mediated phosphorylation are both crucial for stabilizing KDM1A, suggesting the potential of targeting the CDK9-RNF20-KDM1 axis in cancer therapy (Fig. 6) [93].

KDM2A is intimately related to cancer-associated fibroblasts (CAFs) formation. Breast cancer cells secrete IL-6 to induce overexpression of KDM2A via a novel STAT3/NFκB p50 axis in CAFs. The high level of KDM2A in CAFs mediates the secretion of CXCR2-associated chemokines, promoting M2 macrophage polarization [306]. Additionally, KDM2A activates fibroblasts, promoting their surface expression of PD-L1, modulating the immune checkpoint regulation of breast cancer cells (Fig. 6) [270].

KDM4A is a key epigenetic silencer of TRAIL and its receptor death receptor 5 (DR5), which are important targets in immunotherapy. By restricting their expression, KDM4A significantly undermines the therapeutic efficacy of TRAIL combined with DR agonistic antibodies. These findings suggest that targeting KDM4A may represent a critical strategy for enhancing antitumor therapy [43].

In pan-cancer analysis, KDM5 mutations are associated with enhanced tumor immunity through increased tumor immunogenicity and enriched immune cells infiltration, suggesting that KDM5 mutations serve as a marker of favorable prognosis for patients [307]. Stimulator of interferon genes (STING) agonists are novel cancer immunotherapy agents that have been tested in multiple clinical trials, and DNA sensing via the cGAS-STING pathway is critical for cancer immunosurveillance [61]. While, STING is often expressed at low levels in various tumors, limiting the application of agonists. Research has shown that KDM5B and KDM5C epigenetically suppress STING expression. Moreover, reactivation of STING expression through KDM5 inhibition induces a robust interferon response dependent on cytosolic DNA in breast cancer cells [62]. The studies above indicate that KDM5B inhibitors enhance tumor immune responses. However, KDM5B also acts as an antigen to activate CD8 + T cell responses, which is associated with better immune responses [308]. Meanwhile, KDM5C modulates immune responses by promoting the expression of inflammatory genes, including *IL1A*, *IL6*, *CXCL1*, *CXCL2*, *CXCL3*, and *CXCL8* [68]. This dual role suggests that KDM5 holds potential for both inhibition and activation in breast cancer immune regulation.

Tumor-associated macrophages (TAMs), which are pivotal components of the tumor microenvironment, are polarized into either proinflammatory M1 or anti-inflammatory M2 subtypes, with M2 polarization is usually linked to a poor prognosis [309, 310]. KDM6B orchestrates a dual regulatory role in macrophage polarization by promoting M1 states [84] and suppressing M2 states [83], thereby reprogramming the tumor microenvironment to enhance antitumor immune responses. KDM6B drives M1 polarization by enhancing the production of proinflammatory cytokines IL-6, IL-1β, and TNF-α, while cell-derived exosomal miR-138-5p targets KDM6B to suppress its activity. [84]. Conversely, reduced KDM6B expression triggers the M2 phenotype through β-catenin/c-Myc pathway activation [83]. In addition, KDM6A loss mediates TGFβ extracellular secretion, thereby inhibiting the expression of cytotoxic genes in CD8 + T cells and exerting immune-suppressive effects [75]. Overall, both KDM6A and KDM6B have a positive impact on tumor immune regulation.

### Drug resistance

#### Endocrine therapy resistance

The sustained or ligand-independent activation of the ER is a pivotal factor in endocrine therapy failure. Epigenetic modifications represent a significant mechanism underlying this activation [311]. The KDM family, by modulating histone methylation at transcription factor regions, regulates transcriptional activation or repression. In breast cancer, certain KDM members, including KDM1, KDM3, KDM4 act as ERα co-activators and playing a crucial role in endocrine resistance [8, 9, 38, 91].

ERα co-regulator PELP1 interacts with KDM1 to strengthen ERα transcriptional control. Drug sensitivity studies indicate that PELP1 siRNA and KDM1 inhibitors can significantly reduce tumor size and inhibit cell proliferation [8]. Additionally, PELP1-KDM1 signaling positively regulates the HER2-aromatase pathway, increasing aromatase expression and contributing to aromatase inhibitor (AI) resistance. Therefore, KDM1 is pivotal in breast cancer endocrine resistance and targeting the PELP1-KDM1 axis can effectively restore treatment sensitivity and represents a novel strategy for breast cancer patients (Fig. 7) [9].

In HER2-positive breast cancer, ACK1 phosphorylates and activates KDM3A, enabling KDM3A to activate the expression of HOXA1, a pro-oncogenic downstream target of ERα, in an estrogen-independent manner. This ACK1-KDM3A-HOXA1 signaling axis has been identified as a critical mediator that enables HER2-positive breast tumors to circumvent endocrine sensitivity, thereby driving the acquisition of Tamoxifen resistance (Fig. 7) [38].

KDM4B binds to the ERα activation function 1 (ERα AF1) domain and interacts with steroid receptor coactivator 3 (SRC-3) and other transcriptional activators to facilitate ERα transcriptional activity. During endocrine therapy, SRC-3 dissociates from ERα AF2 in competition with TAM. Remarkably, KDM4B degradation is critical for preventing SRC-3 off the AF2. In absence of Fbxo22, the E3 ligase responsible for KDM4B degradation, KDM4B remains stable and collaborates with SRC-3. Thus, KDM4B enhances transcriptional activity of ERα via interaction with the AF1 domain in a ligand-independent manner, resulting in Tamoxifen resistance (Fig. 7) [91].

KDM5, which functions as a H3K4 demethylase to exert transcriptional repression also contributes to endocrine resistance by recruiting other transcriptional activators through ERα-mediated transcriptional activation [49, 66]. KDM5C directly binds to ERα and interacts with the P-TEFb/ZMYND8 complex at ERα enhancer regions, thereby promoting the transcriptional activation of ERα downstream target genes (Fig. 7) [66]. Additionally, KDM5A cooperates with the histone acetyltransferase p300 to enhance the expression of ERα target genes, such as *NRIP1* and *CCND1*, consequently promoting the proliferation and selective ER modulator (SERM) insensitive in breast cancer cells (Fig. 7) [49]. Studies on the application of KDM5 inhibitors have revealed that short-term treatment with the KDM5 inhibitor C70/C49 reduces intracellular ERα protein levels. However, in KDM5 inhibitor-resistant cells (KDM5IR), ERα protein levels are comparable to those in parental MCF7 cells. Moreover, KDM5IR cells show the promotion of cell proliferate without estrogen and exhibit higher ERα phosphorylation levels after E2 treatment than that in MCF7 cells (Fig. 7) [52]. These findings indicate that KDM5 as a potential factor in ER + breast cancer is related with ERα signaling pathway.

KDM5 regulates the crosstalk between multiple signaling pathways [49]. Notably, KDM5 partners with ER, NRIP1, and HDAC1, employing its demethylase action to suppress IGFBP4 and IGFBP5. This action alleviates the inhibition of IGF1R, thereby activating the IGF1R signaling pathway. Additionally, KDM5 stabilizes EGFR and HER2 proteins in a demethylase-independent manner, enhancing their activity and subsequently activating the ErbB signaling pathway. Through demethylase-dependent and -independent mechanisms, KDM5 activates IGF1R and ErbB signaling crosstalk, which further stimulates the PI3K/AKT pathway[56]. Further research confirms that combining TAM with a PI3K/AKT inhibitor (BKM120) such as BKM120, can effectively counteract drug resistance caused by KDM5 [49].

Single-cell analysis shows endocrine resistance mainly results from selecting pre-existing genetically distinct cell populations [52]. KDM5 increases transcriptomic heterogeneity in tumor cells, creating more phenotypically diverse cells, some intrinsically resistant to endocrine therapy. Knocking out KDM5A not only reduces the number of resistant cells but also promotes the expression of downstream tumor-suppressor genes P21 and BAK1, thus enhancing tumor cell sensitivity to endocrine therapy [52]. In breast cancer, Myc and TFAP2C, downstream ER target genes and transcription factors, form a complex with KDM5B at the CDKN1A promoter. This interaction impacts CDKN1A expression, inhibiting cell cycle arrest and allowing cells to keep proliferating under endocrine therapy [56].

#### Trastuzumab resistance

Among breast cancer subtypes, HER2-positive cases account for 15–20% [312]. Trastuzumab as an anti-HER2 monoclonal antibody binds to HER2's extracellular domain to block downstream signaling, significantly improving the patient survival. However, trastuzumab resistance remains a challenge, with mechanisms including HER2 mutations and alternative pathway activation [313]. Growing evidence highlights the role of epigenetic mechanisms, including the KDM family members that are emerging as therapeutic targets in breast cancer. KDM inhibitors, when combined with trastuzumab, show promise in overcoming resistance and improving outcomes for HER2-positive breast cancer patients [81, 86, 110].

In HER2-positive breast cancer cell lines, KDM5 inhibitor (KDM5-Inh1) shows synergistic effects when combined with trastuzumab or lapatinib. Specifically, in induced-resistant cell models (e.g., BT-474/TrastR, SK-BR-3/TrastR, and SK-BR-3/T100), KDM5-Inh1 still inhibits cell proliferation, suggesting KDM5 inhibitors may overcome trastuzumab resistance [110].

In trastuzumab-resistant cells and patient samples, tRF-27 is significantly upregulated, with its expression level serving as a potential biomarker to predict trastuzumab tolerance [314]. KDM6A is also upregulated in trastuzumab-resistant breast cancer cells, reducing histone H3K27me3 levels on the promoter region of tRNA-Cys-GCA gene to activate its transcription, thereby increasing tRF-27 accumulation. Thus, GSK-J4 as the KDM6A inhibitor can inhibit tRF-27 production and restore trastuzumab sensitivity [81].

In HER2-positive breast cancer, KDM7B is overexpressed and functions as a transcriptional coactivator, collaboratively enhancing the HER2 signaling pathway. Specifically, KDM7B increases *IL-6* gene transcription, thereby activating the STAT3 signaling pathway. This activation triggers alternative growth pathways to promote tumor cell survival and proliferation, ultimately leading to trastuzumab resistance [86]. Briefly, targeting KDM5, KDM6, and KDM7 with specific inhibitors, either alone or in combination with trastuzumab, represents a novel and potentially effective approach to overcome treatment resistance in breast cancer.

#### Chemotherapy resistance

In TNBC, treatment choices are extremely limited due to the absence of common therapeutic targets, ER, PR and HER2. Presently, anthracyclines and taxanes are standard first-line chemotherapies for TNBC. However, most patients develop resistance to these drugs, leading to a significant decline in treatment effectiveness and endangering their health [315]. KDM family as a key group of epigenetic regulators, plays an essential role in chemotherapeutic efficacy by finely modulating downstream genes that influence several critical tumorigenesis-related pathways.

The chemoresistance in breast cancer is closely associated with cancer stem cells (CSCs). The inherent resistance of CSCs to chemotherapy enables them to persist during treatment, expand subsequently, and thereby trigger tumor recurrence and chemoresistance. Moreover, chemotherapy drugs may themselves boost the stemness of CSCs, further increasing their resistance [73, 76].

In cancer therapy, interferon-I (IFN-I) can activate anti-tumor immunity, while it potentially drives tumor cell progression in some cases. In breast cancer, IFN-I promotes cancer cell reprogramming by activating KDM1B. KDM1B boosts the expression of pluripotency genes like Nanog, Sox2, and Oct4, giving the cells stem cell-like properties. Drug tests show cells overexpressing KDM1B are more resistant to chemotherapeutic agents such as doxorubicin (DOX) and oxaliplatin (OXP). Conversely, KDM1B knockdown increases cell sensitivity to these drugs [117].

The poised state, an epigenetic mechanism marked by both activating (H3K4Me3) and repressive (H3K27Me3) histone modifications, keeps genes in a standby mode for rapid transcriptional responses [316, 317]. In breast cancer, chemotherapeutic agents can activate these poised oncogenes, conferring drug resistance. KDM5C plays a role in repressing these genes. Mechanistically, ZMYND8, a potential tumor-suppressing chromatin reader, recruits KDM5C and EZH2 to inhibit the expression of these poised oncogenes, thereby reducing doxorubicin-induced tumorigenicity and drug tolerance [67].

KDM7A influences *MKRN1* expression, which is closely associated with cell migration, apoptosis, and invasion, via H3K27me3 demethylation, thereby modulating the biological behavior in TNBC cells. Compound 4, a potent KDM7A inhibitor, directly binds to KDM7A and inhibits its interaction with methylated histones. This action suppresses MKRN1 transcription and reduces the expression of stem cell markers CD133, CD44, and ALDH1A1 while increasing the levels of cell cycle regulators p27, p21, and p16. As a result, compound 4 significantly inhibits TNBC taxol-resistant cells [87].

OCT4, a marker of pluripotency, is a key transcription factor regulating embryonic development and the behavior of cancer stem cells [318]. Paclitaxel induces S100A10 expression, which forms a complex with ANXA2, interacting with histone chaperone SPT6 and histone demethylase KDM6. The S100A10/ANXA2/SPT6/KDM6A complex is recruited to OCT4-binding sites, modulating downstream pluripotency-related genes (*NANOG*, *SOX2*, *KLF4*). KDM6A removes H3K27me3 marks, activating transcription and enriching BCSCs [76]. Chemotherapy-induced A2BR expression activates the p38MAPK pathway, causing nuclear translocation of chromatin remodeler SMARCD3, which interacts with KDM6A and histone acetyltransferase p300, recruiting them to pluripotency-related genes. KDM6A removes H3K27me3 while p300 adds H3K27ac, enhancing FOXO3 binding and transcriptional activation of expression of these pluripotency factors, thereby contributing to the maintenance of cancer stemness [73]. These findings indicate that KDM6A plays a critical role in chemotherapy-induced cancer stem cell enrichment.

In addition to stemness, multiple biological mechanisms affect chemotherapeutic sensitivity, including apoptosis and cell cycle. Notably, the role of individual KDM members is not absolute, for example, KDM1 can participate in resistance and also maintain sensitivity [13, 319]. Thus, the impact of KDMs on chemoresistance is multifaceted and involves diverse pathways.

KDM1A appears to be linked to chemoresistance, as its dissociation from chromatin is associated with maintaining chemosensitivity in ER + breast cancer. KDM1 interacts with SIN3A/HDAC to jointly regulate genes such as *CASP7*, *TGFB2*, *CDKN1A* (*p21*), *HIF1A*, *TERT*, and *MDM2*, which are closely linked to cell apoptosis, proliferation, and migration. When treated with the chemotherapeutic drug CPT (camptothecin), KDM1A and SIN3A/HDAC dissociate from the promoters of p21 and TGFB2, increasing their expression to induce cell cycle arrest and apoptosis, thereby enhancing chemosensitivity [13].

IL-6 from cancer cells activates STAT3 and NFκB p50, which boost KDM2A expression. High KDM2A levels enhance cancer-associated fibroblast (CAF) function, driving secretion of CXCR2-related chemokines (CXCL2, CXCL5, IL-8). This promotes M2 macrophage polarization and CCL2/CCR2 signaling, which can increase paclitaxel (PTX) resistance by creating an immunosuppressive microenvironment and promoting tumor cell survival [306]. Additionally, KDM3A induces chemoresistance by demethylating p53 and inhibiting its transcriptional activity. Specifically, KDM3A knockout significantly heightens breast cancer cell sensitivity to chemotherapeutic agents like paclitaxel (PTX) and cisplatin (CDDP) via enhancing chemotherapy—induced apoptosis [39].

BRD4 is an epigenetic factor that activates gene expression by interacting with super-enhancers or recruiting the P-TEFb complex, thereby promoting tumorigenesis [320, 321]. In breast cancer, BRD4 colocalizes with the KDM1/NuRD transcriptional repression complex, where KDM1 is crucial for the function of BRD4 inhibitors (JQ1) [322, 323]. JQ1 reduces BRD4 binding to chromatin, suppressing chemotherapy resistance-related genes such as GNA13 and PDPK1 through KDM1/NuRD mediation. However, long-term JQ1 use can lead to resistance due to PELI1-mediated KDM1 ubiquitination and degradation. The study suggests combining JQ1 with PELI1 inhibitors to maintain KDM1 stability, which is key to inhibiting resistant gene expression and preserving tumor cell chemosensitivity [319].

In MCF10A cells, KDM6A loss leads to chemoresistance to multiple agents. CRISPR/Cas9-mediated KDM6A knockout decreases sensitivity to paclitaxel, AZD2014, and dasatinib. Mechanistically, KDM6A loss activates TGFβ signaling, which is a key driver of chemoresistance [75].

Furthermore, cancer chemoresistance primarily driven by the activity of ATP-binding cassette (ABC) transporters, which actively efflux chemotherapeutic agents from cancer cells, reducing their intracel lular concentrations and therapeutic efficacy for cancer. Recent studies have highlighted the pivotal role of long noncoding RNAs (lncRNAs) and histone methylase EZH2 in chemoresistance by modulating the expression and activity of ABC transporters [324]. It suggests that certain epigenetic factors may modulate ABC transporter expression via chromatin modifications, contributing to multidrug resistance.

#### CDK4/6 inhibitor resistance

CDK4/6 inhibitors have demonstrated significant therapeutic efficacy in ER +/HER2 − breast cancer and have received approval for clinical use [325, 326]. Research discovers a molecular switch between MLL1-Menin and MLL3/4-UTX at CDK inhibitor gene promoters CDKN2C. At basal conditions, Menin bound to the promoters to repress them, while Menin-MLL inhibitor impedes Menin from the promoter, thereby enabling MLL3/4-UTX to reactive CDKN2C. Moreover, UTX loss causes resistance to Menin–MLL inhibitor, which can be overcome by combining with CDK4/6 inhibitors to augment therapy response [327]. Additionally, analysis via the LINCS database shows that gene expression changes induced by KDM6B inhibitors (GSK-J4) closely resemble those caused by CDK4/6 inhibitors (palbociclib), suggesting that KDM6B promotes an oncogenic CDK4/6-pRB-E2F pathway. While in breast cancer, overexpression of CDK4/6 or Rb1 knockout conferred resistance to both palbociclib and GSK-J4. This indicates that CDK4/6 inhibitor resistance cells also exhibit reduced responsiveness to GSK-J4, despite they share synergistic effects at the transcriptomic level [328]. Although CDK4/6 inhibition can compensate for tumor resistance caused by KDM6A loss, KDM6B inhibitors are not effective in overcoming resistance to CDK4/6 inhibitors. The challenge of defeating CDK4/6 inhibitor resistance remains to be addressed.

#### PI3K inhibitor resistance

In breast cancer, PIK3CA gene mutations often lead to abnormal PI3K pathway activation, driving tumor cell growth, anti-apoptosis, and treatment resistance across multiple subtypes. The emergence of PI3K inhibitors offers new hope for patients with PIK3CA mutations who are resistant to endocrine therapy and HER2-targeted trastuzumab [329–331]. In the luminal subtype, KDM6B promotes IGFBP5 expression, which confers protection against PI3K/AKT inhibitor-induced apoptosis and thereby mediates resistance to PI3K inhibitors [79].

Meanwhile, the effectiveness of PI3K inhibitors in TNBC is limited, highlighting a critical need for improved strategies against this aggressive, target-lacking tumor. KDM family members exhibit functional involvement in PI3K inhibitor treatment for breast cancer and may serve as targets to improve efficacy and overcome drug resistance [180, 193]. The presence of KDM5C significantly enhances the therapeutic effect of PI3K inhibitors. Buparlisib and vitamin C work synergistically, promoting the nuclear translocation of KDM5A. Vitamin C also enhances the demethylation activity of KDM5A, reducing H3K4me3 levels at the promoter regions of several PI3K pathway genes including AKT2, mTOR, GSK3α, and mLST8. This reduction inhibits the expression of these PI3K-related genes. This synergistic mechanism greatly improves the therapeutic effect of buparlisib on TNBC cells, presenting a novel strategy for clinical application [193].

PTEN is a key tumor suppressor that encodes a protein managing intracellular signaling, particularly the PI3K-AKT pathway [332]. Its loss in breast cancer leads to abnormal PI3K-AKT activation and resistance to PI3K inhibitors. Single-agent PI3K inhibitors are less effective in PTEN-deficient tumors. However, combining them with KDM4B inhibitors enhances antitumor efficacy. KDM4B inhibition activates the UPR pathway to induce apoptosis. This synergistic effect offers a new strategy for treating PTEN-deficient TNBC [180].

## Novel Inhibitors Targeting KDMs

Epigenetic therapies have emerged as a promising frontier in cancer therapeutics [333]. Given the critical role of the KDM family in regulating various biological processes in the breast that are closely associated with tumorigenesis and progression, and the fact that KDMs often act as oncogenic factors in these contexts, the development of specific KDM inhibitors has become increasingly important and attractive as a therapeutic strategy in cancers [334–336]. Recent studies focus on small-molecule inhibitors targeting KDMs, which can be categorized based on their mechanisms of action into enzyme activity-targeting inhibitors and non-enzymatic competitive inhibitors [337]. The clinical development landscape for KDM inhibitors has predominantly targeted histone lysine demethylases 1–7 (KDM1-7) [338–340]. We summarized KDM inhibitors that exhibit tumor-suppressive effects in breast cancer and enhance therapeutic efficacy when combined with existing drugs, highlighting their potential as promising therapeutic strategies (Table 3).

### KDM1A (LSD1) inhibitors

The oncogenic role of KDM1A across various molecular subtypes of breast cancer highlights the clinical potential of selective KDM1A inhibitors through enzymatic blockade, with emerging evidence positioning KDM1A inhibition as a promising therapeutic frontier [338, 341]. Iadademstat (ORY-1001) is a highly selective and potent covalent inhibitor of LSD1, targeting FAD-binding catalytic domain called amine oxidase-like domain (AOD) (Fig. 1). ORY-1001 attenuates breast cancer stem cell properties through subtype-specific modulation of core stemness regulators. In luminal B and HER2 + variants, the compound targets the SOX2 enhancer domain to block associated stemness maintenance programs [103]. Mechanistically, the RBBP7 super-enhancer complex recruits KDM1A to epigenetically activate a pluripotency network covering SOX9, SOX2, OCT4, CCND1, thereby driving malignant stem cell expansion. Preclinical validation confirms efficacy of ORY-1001 in disrupting this oncogenic axis across both luminal and TNBC models [104].

Multiple KDM1A inhibitors enhance antitumor immunity, including in breast cancer [106]. GSK2879552 covalently binds to the FAD cofactor in the catalytic site of KDM1A, leading to irreversible inactivation of its demethylase activity. Studies in melanoma and leukemia cells demonstrate that GSK2879552 and ORY1001 increase the secretion of multiple cytokines by T cells, including IL-2, TNFα, IFNγ and enhancing their cytotoxic function. Furthermore, these inhibitors synergize with immune checkpoint blockade (ICB) to augment antitumor efficacy [105]. Similar effects have been validated in TNBC. HCI-2509 as the reversible KDM1A inhibitor targeting TOWER domain of KDM1A significantly suppress tumor growth and lung metastasis when combined with anti-PD-1 antibodies. These effects are accompanied by increased infiltration of CD8 + T cells in the tumor microenvironment [107]. Collectively, these findings highlight that KDM1A inhibitors as immunotherapeutic adjuvants to reverse immune suppression in breast cancer.

### KDM4 inhibitors

KDM4 is frequently associated with breast cancer progression, and inhibitors targeting KDM4 have been investigated for their potential in antitumor therapy [108, 167]. NCDM-32B targeting the JmjC catalytic domain of the KDM4 sub-family is a potent and selective inhibitor of the KDM4. In basal-like breast cancer, NCDM-32B affects critical pathways related to tumor growth, including DDR and cell cycle progression [109]. QC6352 as a selective inhibitor of KDM4A targeting the JmjC catalytic domain reduces EGFR expression to inhibit the proliferation and self-renewal capacity of BCSCs. This mechanism leads to suppression of TNBC cell growth. Notably, QC6352 demonstrates significant antitumor activity in breast cancer xenograft models without causing obvious side effects [108]. In HER2-positive breast cancer leptomeningeal carcinomatosis (HER2 + LC), KDM4A and KDM4C are significantly overexpressed and promote tumor cell proliferation through an autocrine GMCSF/GMCSFRα signaling pathway. JIB-04 is a cell-penetrant, pan-selective JmjC inhibitor whose primary efficacy against KDM4A and KDM4C is mediated through binding inside the JmjC catalytic domain. JIB-04 directly suppresses GMCSF-dependent proliferation by reducing GMCSF expression. Additionally, JIB04 remodels the epigenetic landscape of cancer cells by increasing H3K9me3 and H3K36me3 levels, further inhibiting tumor progression. This dual mechanism highlights JIB04 as a potential therapeutic strategy for HER2 + LC [42]. Compound 4 (C-4) is a competitive inhibitor of KDM4A and KDM4B that occupies the JmjC catalytic domain. C-4 strongly induces the expression of TRAIL and its receptor DR5 in various cancers, including breast cancer, thereby activating TRAIL-dependent apoptotic pathways. C-4 exhibits robust antitumor activity in both TRAIL-sensitive and -resistant cancer cells in vitro and suppresses tumor growth in xenograft models. Furthermore, C-4 synergizes with ONC201 (a TRAIL inducer) to enhance tumor sensitivity to ONC201, underscoring its potential as an effective adjuvant [43].

### KDM5 inhibitors

In breast cancer research, KDM5 demethylases have been extensively characterized as context-dependent regulators, suppressing or promoting breast cancer progression through distinct signaling [339]. Different KDM5 inhibitors have been found to effectively inhibit breast cancer both in vitro and in vivo experiments. However, their development remains challenging due to the dual roles of KDM5.

Pharmacological inhibition of KDM5 elevates genome-wide H3K4me3 deposition, which epigenetically alter transcriptional regulation and tumor cell heterogeneity [52, 111]. Monotherapy with the pan-KDM5 inhibitor CPI-455 targeting JmjC catalytic domain exerts limited transcriptional modulation in luminal breast cancer. However, when combined with the DNA demethylating agent 5-aza-2’-deoxycytidine (DAC), it significantly enhances DAC-induced transcriptional activation, particularly reactivating tumor-suppressive genes such as TP53INP1 and IRF2BP2. Functionally, CPI-455 and DAC together demonstrate a pharmacological synergistic effect to suppress breast cancer progression [111]. Research shows that H3K4me3 promoter modifications correlate positively with transcriptional consistency. KDM5 inhibitor treatment reduces transcriptional heterogeneity in tumor cells, especially in ER-positive breast cancer. Moreover, combining the selective estrogen receptor downregulator fulvestrant with small-molecule inhibitors of KDM5 (KDM5-C49 or KDM5-C70) targeting JmjC catalytic pocket enhances endocrine therapy sensitivity in resistant cell lines (FULVR and TAMR). This strategy of reducing tumor heterogeneity to improve endocrine therapy sensitivity may offer a new therapeutic approach (Fig. 7) [52].

In HER2 + cell lines BT-474 and SK-BR-3, KDM5 inhibitors synergize with HER2-targeted therapies. The pan-KDM5 inhibitor KDM5-inh1 modulates the expression of downstream genes in the HER2 signaling pathway, thereby increasing tumor sensitivity to HER2-targeted drugs such as trastuzumab and lapatinib [110]. Several KDM5 inhibitors, including KDM5-C70, Dong-A-167, GDC-50, and CPI-48, increase H3K4me3 levels at the STING gene promoter, activating STING expression and enhancing antitumor immune responses. Combination strategies with STING agonists can restore sensitivity in immunotherapy-resistant patients [62].

### Inhibitors targeting for KDM6 or KDM7

Growing evidence shows that the aberrant activity of KDM6A and KDM6B is associated with certain cancer progression by activating different transcription factors via numerous signaling pathways [342]. The KDM6 inhibitor GSK-J4 targeting JmjC catalytic pocket demonstrates potent therapeutic efficacy in breast cancer models, suppressing tumor progression through epigenetic modulation of H3K27me3-mediated oncogenic pathways. In the MDA-MB-231 and MCF7 cell lines, GSK-J4 markedly reduces the number and size of breast cancer stem cells (BCSCs) [112]. Moreover, GSK-J4 acting as KDM6 inhibitor can be used alone or in combination with other anticancer drugs (e.g., trastuzumab) to enhance therapeutic efficacy [81].

Compound 4 identified as a potential KDM7A inhibitor by occupying the JmjC catalytic domain shows promise in TNBC treatment [87]. MKRN1, a key downstream gene of KDM7A, is overexpressed in TNBC and linked to chemoresistance. Compound 4 suppresses MKRN1 transcription to induce G1-phase cell cycle arrest and reduce TNBC cell stemness. It exhibits significant antitumor activity in both paclitaxel-resistant and -sensitive TNBC cell lines, suggesting it can overcome chemoresistance and offers a novel strategy for TNBC treatment [87].

Above all, these studies illustrate that KDM inhibitors offer novel targets for tumor therapy. However, KDM inhibitor resistance may be a challenge event in the future. The studies suggest that KDMs commonly associate with different protein complexes with altered roles in the context of complexes. The function of each demethylase can be changed with the alteration of associated chromatin complex. This association may be one reason for the difficulty in targeting these enzymes for the desirable outcome or KDM inhibitor resistance. Increased KDM4A binding at specific loci associated with replication machinery, DNA re-replication, and transient site-specific ecDNA copy gains (TSSGs). This process occurred at regions commonly amplified in cancers and is associated with drug resistance and proliferation in cancer [343].

Thus, future efforts should focus on the development of small molecules or other strategies to target this enzyme and/or the complexes associated with KDMs to get optimal impact while minimizing off-target effects and toxicity.

### KDM inhibitors in clinical implications

Having established the molecular mechanisms and biological functions of histone demethylases in breast cancer, what is the current status of KDM inhibitors in clinical trials for breast cancer? Therefore, this section will focus on the translatable findings for KDM family enzymes in clinical trials, clinical pharmacology [344], and implications, although numbers of studies for KDM enzymes are mainly in pre-clinical periods for breast cancer. To date, the clinical trials for KDM1A, KDM4, and KDM5 inhibitors are launched.

KDM1A (LSD1) inhibitors can be grouped in covalent and non-covalent agents. Until now, a number of LSD1 inhibitors have entered clinical trials for hematological and/or solid cancers [345]. Having established the studies of clinical trials with LSD1 inhibitors for solid tumors, such as Ewing sarcoma (EWS) and neuroendocrine tumors (e.g., NCT03600649, NCT03895684, and NCT05420636) [346]. In NCT03895684, seclidemstat acting as an LSD1 inhibitor demonstrated positive activity among advanced sarcoma patients with a manageable safety profile. However, in NCT02034123, GSK2879552 as a selective inhibitor of LSD1 provided poor disease control and caused high toxicity rate in patients with small-cell lung cancer, leading to the study to be terminated [347]. To date, there is no clinical trials for LSD1 inhibitor in breast cancer yet. Much of the current studies suggests that the promotion function of LSD1 on cancer progression are multifaceted, and future efforts should focus on the development of small molecules or other strategies to target LSD1 itself and/or the protein complexes interacted with it.

KDM4 enzymes are highly expressed and amplified across different subtype of breast cancer and play crucial roles in driving tumor growth. These enzymes therefore appear to be promising therapeutic targets, and numerous chemical inhibitors have been developed. Until now, TACH101 targeting KDM4 isotypes (A-D) inhibitor is the JmjC demethylase inhibitor being tested as a single agent in phase 1 clinical trials in patients with advanced and metastatic tumors (NCT05076552). TACH101 exerted impressive efficacy in inhibiting proliferation and growth of tumors in several xenograft models in vivo and patient-derived organoid models across multiple tumor types [348]. These studies make a strong augment for the importance of developing more therapeutic agents targeting KDMs for breast cancer treatment.

KDM5B/JARID1B is a crucial regulator of cancer stemness and lineage specification in a number of cancers such as breast cancer. KDM5B is found mutated in breast cancer and that it modulates luminal and basal cell-specific expression programs [54]. Until now, the first clinical trial targeting JmjC demethylase activity of KDM5 enzymes was launched in 2016, investigating the efficacy of the GS-5801 (KDM5 inhibitor) in chronic hepatitis B, however unsuccessful in its application for chronic hepatitis B [349]. Thus, the clinical applications for KDM5 inhibitors is highly relevant for cancer and deserves deeper evaluation.

**Table 3:** The treatment effects of KDM inhibitors in breast cancer

KDMs	Inhibitors	BC subtypes	Treatment effects	Experimental Model	Ref
KDM1	ORY-1001	Luminal, HER2 +	Targetes SOX2-driven breast cancer stemness	Patient-derived xenograft (PDX) model	[103]
	ORY-1001	Luminal, TNBC	Inhibits cell metastasis and stemness in RBBP7 highly expressed breast cancer cells	PDX model, Lung metastasis model	[104]
	GSK2879552, ORY1001	Non-BC	Activates CD8 + T cells to enhance antitumor immunity	Syngeneic mouse tumor model, Humanized mouse model (NCG)	[105]
	HCI-2509, tranylcypromine	TNBC	Enhances the inhibitory effect of anti-PD-1 antibody on TNBC proliferation and pulmonary metastasis	Syngeneic mouse tumor model	[106, 107]
KDM4	QC6352	TNBC	Downregulates EGFR expression in tumor stem cells to suppress tumor growth and metastasis	PDX model	[108]
	JIB04	HER2 +	Downregulates GMCSF expression to inhibits proliferation of HER2-positive leptomeningeal carcinoma cells	PDX model	[42]
	Compound 4	TNBC, Non-BC	Enhances sensitivity to the TRAIL agonists ONC201 via upregulating expression of TRAIL and DR5	Cell line-derived xenograft (CDX) model	[43]
	NCDM-32B	TNBC	Inhibits cell proliferation of basal breast cancer cell lines	Cell lines	[109]
KDM5	KDM5-C70, Dong-A-167 GDC-50 CPI-48	Luminal, HER2 +	Combines with STING agonists to synergistically enhance antitumor immunity	Bioinformatics analysis (TCGA, TIMER)	[62]
	KDM5-C49, KDM5-C70	Luminal	Combines with fulvestrant to enhance the efficacy of endocrine therapy	CDX model	[52]
	KDM5-inh1	HER2 +	Combines with HER2-targeting agents and remains sensitive in trastuzumab-resistant cells	Cell lines	[110]
	CPI-455	Luminal	Enhances deoxycytidine-mediated transcriptional activation	CDX model	[111]
KDM6	GSK-J4	TNBC	Inhibits cell stemness in breast cancer cells	CDX model	[112]
	GSK-J4	HER2 +	Reduces tRF-27 levels to enhance trastuzumab sensitivity in cells	CDX model	[81]
KDM7	Compound 4	TNBC	Inhibits MKRN1 transcription to inhibit tumor progression in paclitaxel-resistant cells	Cell lines	[87]

## Conclusions and perspectives

Breast cancer remains a significant global health challenge due to its heterogeneity and complex molecular mechanisms. The histone lysine demethylases (KDMs) family, with its crucial roles in modulating gene expression and chromatin dynamics, has emerged as a key player in breast cancer progression, metastasis, and therapeutic resistance. This comprehensive review has elucidated the molecular mechanisms and biological functions of various KDM family members in breast cancer, highlighting their potential as therapeutic targets.

The KDM family, categorized into seven subfamilies (KDM1-7), exhibits diverse functions in breast cancer, ranging from transcriptional regulation and chromatin remodeling to interactions with non-histone proteins. Each subfamily demonstrates distinct roles, with subtype-specific functions in luminal and triple-negative breast cancer (TNBC). For instance, KDM1A and KDM5B play crucial roles in luminal breast cancer by regulating estrogen receptor (ER)-mediated transcription, while KDM4C and KDM6A are more prominent in TNBC through mechanisms related to stemness and metastasis. Additionally, KDMs are implicated in various hallmarks of breast cancer, including DNA damage response, cell cycle regulation, apoptosis, stemness maintenance, metabolic reprogramming, and modulation of the tumor microenvironment. Targeting KDMs represents a promising therapeutic strategy for overcoming drug resistance in breast cancer. Inhibitors targeting KDM1, KDM4, KDM5, and KDM6 have shown potential to enhance the efficacy of endocrine therapy, chemotherapy, and targeted therapy by modulating oncogenic signaling pathways. However, the development of KDM inhibitors faces several challenges such as the dual roles of KDMs in promoting and suppressing tumor progression, KDM inhibitor resistance, and the need for more specific and effective inhibitors targeting enzyme catalytic activity or distinct protein complexes. Future research should focus on several key areas to further advance our understanding and application of KDM-targeted therapies in breast cancer:

- Elucidating Detailed Mechanisms: Further studies are needed to elucidate the detailed molecular mechanisms by which KDMs regulate gene expression, chromatin dynamics, and non-histone protein functions in breast cancer. This includes understanding the interactions between KDMs and other epigenetic regulators, transcription factors, and signaling pathways.
- Subtype-Specific Functions: Given the subtype-specific roles of KDMs, future research should focus on identifying the unique functions and interactions of KDMs in different breast cancer subtypes. This will help in developing more targeted and effective therapies tailored to specific subtypes.
- Overcoming Drug Resistance: Understanding the mechanisms of resistance to KDM inhibitors and developing strategies to overcome this resistance is crucial. This includes exploring combination therapies with other targeted agents, chemotherapy, and immunotherapy to enhance therapeutic efficacy.
- Biomarker Development: Identifying biomarkers that predict response to KDM inhibitors will be essential for personalized medicine approaches. This will help in selecting patients who are most likely to benefit from KDM-targeted therapies and in monitoring treatment response.
- Clinical Trials and Translational Research: Conducting well-designed clinical trials to evaluate the safety, efficacy of KDM inhibitors, and combination treatment strategy in breast cancer patients is critical. Translational research should focus on translating preclinical findings into clinical practice and addressing the challenges of translating basic science discoveries into effective treatments.
- Epigenetic Landscape: Investigating the broader epigenetic landscape in breast cancer, including the interplay between KDMs and other epigenetic modifications such as DNA methylation and non-coding RNA regulation, will provide a more comprehensive understanding of breast cancer biology.

In conclusion, the KDM family members play intricate roles in breast cancer progression and therapeutic resistance. Targeting KDMs offers a promising therapeutic avenue, but further research is needed to fully harness their potential. Future studies should focus on elucidating detailed mechanisms, developing subtype-specific therapies, overcoming drug resistance, and conducting robust clinical trials to bring these promising therapeutic strategies to clinical practice.

## Data Availability

No datasets were generated or analysed during the current study.

## Abbreviations

AR: Androgen receptor
ARID: AT-rich interaction domain
ASXL2: Additional sex comb-like 2
BAER: Basally active estrogen-repressed
BCS: Breast cancer stemness
CAFs: Cancer-associated fibroblasts
CDKs: Cyclin-dependent kinases
CoREST: Corepressor for element-1-silencing transcription factor
CR: Caloric restriction
CSCs: Cancer stem cells
CtBP: Carboxy-terminal binding protein
CTCF: CCCTC-binding factor
DSBs: DNA double-strand breaks
EMT: Epithelial-mesenchymal transition
EpCAM: Epithelial cell adhesion molecule
ER: Estrogen receptor α
EREs: Estrogen-response elements
ERα AF1: ERα activation function 1
ERα AF2: ERα activation function 2
FAD: Flavin adenine dinucleotide
GASC1: Gene amplified in squamous-cell carcinoma
GM-CSF: Granulocyte-macrophage colony-stimulating factor
HDAC1: Histone deacetylase 1
HER2: Human epidermal growth factor receptor 2
HIF-1α: Hypoxia-inducible factor 1-alpha
HR: Homologous recombination
HR: Hormone receptor
ICB: Immune checkpoint blockade
IFN-I: Interferon-I
IGFBP4/5: Insulin-like growth factor-binding protein 4/5
ITGA6: Integrin α6
ITGB1: Integrin β1
JmjC: Jumonji C
KDMs: Lysine demethylases
LRR: Leucine-rich repeat
LSD1: Lysine-specific demethylase 1
m6A: N6-methyladenosine
MMPs: Matrix metalloproteinases
MTA1: Metastasis associated 1
MT-CO2: Mitochondrial Cytochrome c oxidase II
NHEJ: Non-homologous end joining
NRIP1: Nuclear receptor-interacting protein 1
NuRD: Nucleosome remodeling and deacetylase complex
Pan-BC: Pan-Breast Cancer
PDX: Patient-derived xenograft
PELP1: Proline glutamic acid and leucine-rich protein 1
PHD: Plant homeodomain
PR: Progesterone receptor
PRC: Polycomb repressive complex
PTM: Post-translational modification
RBP2: Retinoblastoma binding protein 2
Rg3: Ginsenoside
SASP: Senescence-associated secretory phenotypes
SGOC: Serine-glycine-one-carbon
SMYD2: SET and MYND Domain-Containing Protein 2
SRC-3: Steroid receptor coactivator 3
STING: Stimulator of interferon genes
TAMs: Tumor-associated macrophages
TCA: Tricarboxylic acid
TFs: Transcription factors
TME: Tumor microenvironment
TNBC: Triple negative breast cancer
TOPBP1: DNA topoisomerase 2-binding protein 1
TPR: Tetratricopeptide repeat
UTX: Ubiquitously transcribed tetratricopeptide repeat on chromosome X
YTHDF2: YTH N6-methyladenosine RNA-binding protein 2
ZF-CXXC: Zinc-fnger CXXC
ZHX2: Zinc-fingers and homeoboxes 2
ZMYND8: Zinc finger MYND-type containing 8
